# A Scoping Review of Eating Disorder Clinicians' Experiences, Needs, Views and Wellbeing

**DOI:** 10.1002/jclp.70005

**Published:** 2025-06-16

**Authors:** Kat Novogrudsky, Janet Treasure, Øyvind Rø, Ulrike Schmidt

**Affiliations:** ^1^ Centre for Research in Eating and Weight Disorders King's College London; ^2^ South London and Maudsley NHS Foundation Trust; ^3^ Oslo University Hospital

**Keywords:** burnout, clinician wellbeing, eating disorders, job satisfaction, professional development

## Abstract

**Background:**

Eating disorders (ED) are pervasive and severe mental illnesses affecting up to 15% of females and 5% of males internationally with rates sharply rising in recent decades, especially since the COVID‐19 pandemic. As a result, workload pressures on ED services have surged. The impact of this on ED clinicians and their wellbeing has not recently been investigated. This scoping review examines recent literature on ED clinicians’ experiences, needs, and wellbeing to identify areas for future research and intervention. The goal is to improve clinician support, quality of life, and patient outcomes.

**Methods:**

Following PRISMA guidelines, eight databases and gray literature sources were searched for studies published from 2014 to 2024. Papers were assessed for quality and risk of bias, and mixed‐methods data were analyzed using narrative synthesis.

**Results:**

Sixty‐three studies, encompassing 3,152 multidisciplinary ED clinicians, were included. Clinicians worked across diverse settings with patients of varied presentations. Analyzes suggest that whilst job satisfaction amongst ED clinicians is high and attitudes are generally positive, workplace demands and stressors have a negative impact on clinician wellbeing. Several areas require clearer guidance and further clinician training. Clinicians’ affect is mixed, and an ‘emotional rollercoaster’ is experienced at work. Many clinicians mention a lack of resources as a frustrating obstacle to an optimally operating service.

**Conclusions:**

Clinicians experience working with ED patients as emotionally challenging and occasionally fatiguing, but attitudes are generally positive. However, clinicians are hindered by organizational factors and a lack of resources, including those pertaining to staffing and training.

## 1 Introduction

Eating disorders (ED) are common and complex mental illnesses, characterized by disturbances in eating behaviors, with heterogeneous presentations, ambivalence about treatment, and high risk of relapse(American Psychiatric Association 2013). Internationally, the prevalence and burden of EDs has been rising in recent decades (Wu et al. 2020). During the COVID‐19 pandemic, there has been a further surge of referrals to ED services (Taquet et al. 2022), alongside substantial workforce problems (increases in staff sickness, vacancies and turnover). The ensuing lengthy waitlists and staffing issues may have put a substantial strain on ED clinicians, not to mention the stark impact on patient care.

### 1.1 Challenges to Working With EDs

Aside from these more recent difficulties encountered by ED services, clinicians have described challenges arising from the nature of EDs themselves. Webb et al. (2022) investigated clinicians’ experiences of working with anorexia nervosa (AN), conducting interviews with clinicians delivering inpatient and/or day patient treatment in the UK. Interviews were conducted during the pandemic, though questions were asked about pre‐pandemic service provision. Thematic analysis highlighted several challenges felt by clinicians, including complexities arising from patients’ illness severity, difficult treatment decisions, and limited resources, as well as the need for training and clearer guidance to reduce feelings of uncertainty around their decision making.

### 1.2 Burnout in Healthcare Professionals Working With EDs

Burnout is a state of emotional, physical, and mental exhaustion, which is often characterized by three components: emotional exhaustion, depersonalization, and reduced personal accomplishment (Maslach and Leiter 2006). Emotional exhaustion pertains to feelings of being overwhelmed and exhausted from work demands, depersonalization refers to negative attitudes and feelings towards one's work, and reduced personal accomplishment describes feelings of incompetence or lack of importance at work. Burnout has been associated with negative consequences including poorer physical and emotional health, affecting work and personal functioning (Maslach and Leiter 2008). It is also possible that burnout can impact patient care, such as through impatience or maladaptive non‐verbal communication. Compassion fatigue is another term explored in some literature, referring to clinicians’ exhaustion arising from repeated exposure to patients’ distress (Sorenson et al. 2016). Compassion fatigue is thought to closely mirror posttraumatic stress disorder and may be associated with clinician burnout (Sorenson et al. 2016).

Several factors may increase feelings of burnout in ED clinicians, such as being younger, female or having overweight. Organizational risk factors for burnout include having longer working hours, less experience, insufficient resources and high demands, working in more intensive settings, and experiencing a patient's death (Warren et al. 2012). Some ED‐specific factors may also contribute to burnout – including the nature of EDs (e.g., the chronicity, risk of relapse, and symptom severity), patient characteristics (e.g., ego‐syntonic symptoms, comorbidities), work‐related factors (e.g., lack of resources, poor coordination of care), and poor pay (Warren et al. 2012).

Clinicians may experience feelings of frustration and helplessness whilst working with this patient group (Graham et al. 2020), potentially referring to the recurring and complex nature of the disorder. Other challenges include clinician lack of confidence and knowledge related to ED, negative attitudes, worry, and team issues (Seah et al. 2017; Thompson‐Brenner et al. 2012).

A survey of burnout in ED clinicians found that emotional exhaustion in this group was comparable to that in other mental health professions, whilst levels of depersonalization and reduced personal accomplishment were lower in ED clinicians (Warren et al. 2013). Overall, burnout in ED clinicians was lower than in non‐ED mental health clinicians. These authors suggest that ED clinicians find their work emotionally challenging and at the same time meaningful, valuable, and effective. This potentially explains why levels of burnout were not found to be vastly different across clinician groups. Comparing ED clinicians with and without lived experience, groups did not differ in emotional exhaustion, though lived experience clinicians felt greater personal accomplishment than those without lived experience – meaning they had strong feelings of accomplishment and meaningfulness from their work. The authors suggest that this might be due to lived experience clinicians’ enhanced ability to empathize with patients and to remain optimistic about ED recovery.

Similarly, (Hage et al. 2021) found relatively low levels of burnout in eating disorder clinicians in Norway and noted that ED‐specific factors (e.g., high risk of relapse, somatic complications, patient survival, patient personality perceived to be difficult) and more emotional dissonance (e.g., suppressing emotions to remain neutral or displaying emotions that do not align with one's true feelings) predicted higher levels of burnout in their sample. Such negative work‐related experiences may then detrimentally affect patient care (Garcia and Ramos 2019), necessitating bespoke training for clinicians as well as understandings of their current work‐related needs.

### 1.3 Impaired Clinician Wellbeing and Impact on Practice

Studies have evidenced the impact of clinician burnout on patient safety, such as Garcia (2019), where higher levels of burnout were associated with poorer patient outcomes. In this study, poorer patient safety was reflected by reports of patient dissatisfaction, complaints from patients and families, and medication errors. These authors also found high levels of burnout amongst physicians and nurses, with burnout being influenced by factors such as high workloads and ineffective interpersonal relationships.

Alongside burnout, negative emotions may also influence patient outcomes. Negative emotions are not uncommon amongst clinicians when working with ED patients. Thompson‐Brenner et al. (2012) found that negative clinician reactions towards ED patients commonly included frustration, hopelessness, a feeling of incompetence, and worry. These negative reactions were more frequently held by those with less experience working with ED patients, as experienced psychotherapists did not have such negative attitudes towards this patient group. Greater negative reactions were associated with patients’ lack of improvement and presence of personality pathology, as well as with clinician stigma towards ED and inexperience. Male clinicians also tended to hold more negative attitudes towards ED patients than did female clinicians.

The Cognitive Interpersonal Maintenance Model of AN (Treasure et al. 2011) suggests that there may be various pathways whereby clinician factors might negatively impact on ED treatment. The negative interpersonal factors that may develop include clinicians’ anxiety driving forms of coercion and hostility or alternatively there may be a failure to provide effective treatment by accommodating and accepting AN behavior. Furthermore, especially in in‐patient settings, the clinical environment may encourage rigid rules and striving for perfection which underpin the psychopathology of AN. The isolation from friends, family and education and social opportunities can stunt the development of a non‐AN identity and allow the AN identity to become more fixed (Cardi et al. 2018).

Moreover, an estimated 20‐33% of ED clinicians have had an ED in their lifetime (Warren et al. 2008), signifying the need to understand the impact of clinicians’ own mental health and wellbeing on their practice. Whilst some studies have documented several benefits arising from a clinician's lived experience (Williams and Haverkamp 2015), it is important to recognize the potential impact this may have on a clinician's own wellbeing as well as on their practice.

### 1.4 Research Gap and Current Study

Whilst research exploring the experiences of ED clinicians has been increasing, there remains a lack of comprehensive understanding of their evolving needs and wellbeing—particularly in the wake of the COVID‐19 pandemic and amidst increasing pressures on ED services. Existing reviews, such as Webb et al. (2022), have laid important groundwork by identifying broad themes in intensive ED treatment settings (inpatient and day‐patient). However, these reviews do not fully capture the intensifying challenges across ED settings following the pandemic.

This scoping review sought to build on and expand previous work by offering an updated narrative synthesis, with a specific focus on clinicians’ wellbeing, needs, and perspectives when working with ED patients. This is both timely and necessary, as recent data show alarming rates of psychological distress among NHS healthcare workers, including first‐time suicidal ideation (10%) and suicide attempts (3.9%) during the pandemic period (Padmanathan et al. 2023). Contributing factors include exposure to morally injurious events, lack of managerial support, and reduced ability to provide high‐quality care.

By examining how these factors manifest within ED settings, this review aimed to identify gaps in the current knowledge base, inform future research and clinical interventions, and ultimately contribute to improved support for ED clinicians. A secondary aim was to explore how clinician wellbeing may vary across service types (e.g., inpatient vs. outpatient), patient populations (e.g., comorbid presentations), and clinicians’ own personal or professional histories.

## 2 Methods

### 2.1 Search Terms

The following search terms were used to identify relevant papers: (therapist* OR professional* OR “healthcare worker*” OR clinician* OR “healthcare personnel” OR “health personnel/” or staff) AND (wellbeing OR experienc* OR burnout OR view* OR need* or perspective*) AND (“eating disorder*” OR anorexia nervosa OR bulimia nervosa OR “binge eating disorder” OR “avoidant restrictive food intake disorder” OR “feeding and eating disorder/”).

### 2.2 Information Sources

Eight databases were searched via Ovid: Embase, Ovid MEDLINE, Global Health, APA PsycInfo, HMIC, AMED, PsycArticles, and Social and Public Policy. Additional gray literature databases – OpenDOAR, GreyLit. org, ETHoS – were searched.

### 2.3 Definition of Categories for Inclusion and Exclusion

The SPIDER model was used to support the identification of papers for this review (See Table 1). SPIDER was chosen because it accommodates a wider variety of methodological approaches and data types, including qualitative studies, mixed methods research, and small‐scale or exploratory designs. (Methley et al. 2014).

**Table 1:** SPIDER model of inclusion and exclusion criteria.

SPIDER term	Inclusion criteria	Exclusion criteria
Sample	ED Clinicians, GPs, psychiatrists, psychologists, dietitians, other health professionals involved in ED treatment. Sample includes clinicians from any country.	Clinicians: Dentists, fertility specialists, midwives, alternative medicine therapists, students, school counselors, clinicians who have not directly worked with ED patients. Patient group: Non‐ED patients, drinking, swallowing or feeding disorders patients, weight disorder patients without ED. Non‐clinicians, carers: If paper includes clinician and patient/carer views but results are not segregated by participant group.
Phenomenon of Interest	Experiences working with ED patients, including attitudes and views towards their work, training needs, burnout, and wellbeing. Includes all treatment settings (e.g., inpatient, outpatient, day patient), all ED diagnoses, all patient ages (e.g., child and adolescent, adult, all‐age services).	Clinician views on patient experience, clinician views on new training or treatment modalities, clinician skill development programs, program implementation trials/attitudes or experience with a program, school prevention programs.
Design	All original research studies with empirical methodology, including surveys, interviews/focus groups, Delphi studies. Thematic analyzes. Study must have clear and empirical methodology.	Diagnostic criteria development/standardization, Commentaries, personal accounts, auto‐ethnographies, Systematic reviews, meta‐analyzes, proposed works without data/evaluation, case studies.
Evaluation	Attitudes, views, countertransference, knowledge and confidence, perspectives, willingness, readiness, distress, supervision/support needs, burnout, wellbeing, quality of life, affect, job satisfaction.	
Research Type	Quantitative, qualitative, and mixed‐methods studies.	

### 2.4 Selection Process and Search Strategy

**Figure 1:**
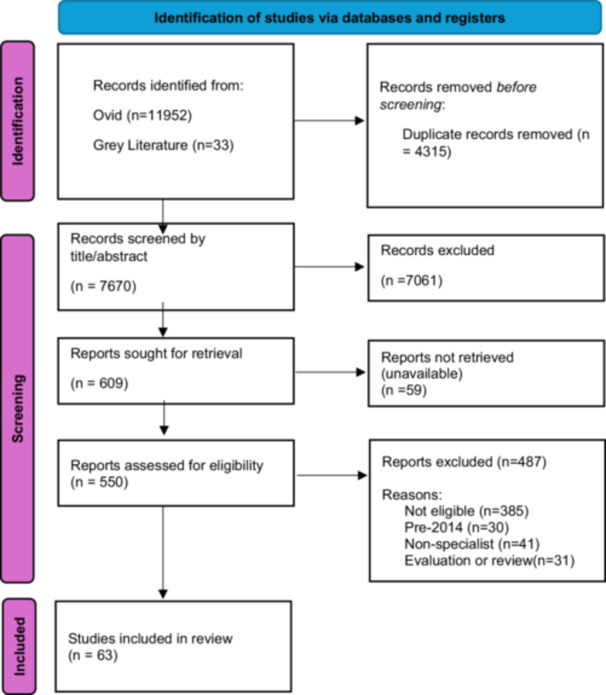
PRISMA flow diagram.

This review was conducted following PRISMA guidelines (see Figure 1; (Page et al. 2021)). The search was run until the end of 2024.

Once all papers were imported into Endnote, duplicates were removed using the deduplication tool, then by hand‐review. Titles and abstracts were first screened. Papers with titles and abstracts which satisfied the inclusion and exclusion criteria were then moved on to full‐text screening.

To maintain a manageable scope and ensure relevance to current clinical contexts, the review was limited to studies published between 2014 and 2024. This 10‐year window reflects a period of significant change in the field, including increased prevalence, public awareness, and shifts in service delivery models for eating disorders. While earlier studies may offer valuable insights, the focus on more recent literature was intended to capture the evolving needs and experiences of clinicians. Papers which fulfilled all criteria then had their data hand‐extracted for analysis by the first author.

Data charting was conducted using a standardized form developed in Microsoft Excel. Extracted data included: author(s), publication year, country, study design, clinician characteristics (e.g., sample size, age, gender, ethnicity, years of experience, personal history of ED), service setting (e.g., inpatient/outpatient/day‐patient/other, adult/child/all age service, public or private), patient characteristics (e.g., diagnosis, comorbidities), quantitative measures, and key findings related to clinician wellbeing, and any practice or policy implications.

Data charting was conducted by the first author. To enhance reliability and rigor, the charting form was piloted on a subset of studies to ensure consistency in extraction. Additionally, the process was iterative and reflexive, with regular revisiting of extracted data to check for accuracy and coherence across studies.

### 2.5 Data Analysis and Synthesis

Quantitative studies were assessed for selection bias, performance bias, detection bias, attrition bias, and reporting bias. Qualitative studies were assessed for credibility, dependability, confirmability, and transferability. A scoring system was created (see Table 2; (Pluye et al. 2009)) and used to categorize risk of bias (low, medium, or high).

**Table 2:** Risk of bias (quality) assessment of included studies (Pluye et al. 2009).

Types of study components	Methodological quality criteria
Qualitative	Clear objective/question Appropriate approach, design or method Description of context, participants and sampling Description of data collection, analysis Description of reflexivity
Quantitative	Appropriate sampling and sample, selection bias Justification of measurements (validity and standards) Control of confounding variables Attrition bias Reporting bias
Mixed Methods	Justification of design Combination of qualitative and quantitative data collection‐analysis techniques or procedures Integration of qualitative and quantitative data or results

A narrative synthesis approach was used to interpret the findings, drawing on principles of thematic analysis. Extracted data were reviewed and coded inductively using Microsoft Excel and NVivo to identify patterns across the studies. Codes were then grouped and refined into broader categories, resulting in the development of ten overarching themes that reflected the key experiences, needs, and wellbeing concerns of clinicians working in eating disorder services. This iterative process involved re‐examining the data to ensure that themes were comprehensive, distinct, and grounded in the included studies. Where available, quantitative findings (e.g., prevalence rates, survey data) were incorporated into the narrative synthesis.

## 3 Results

**Figure 2:**
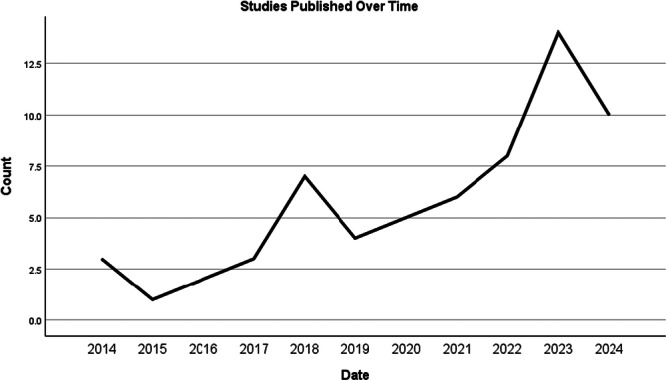
Publication of included studies over time.

Sixty‐three papers were included in the review, comprised of data from 3152 multidisciplinary clinicians working with EDs, including nurses, therapists, general practitioners, occupational therapists, and clinicians with lived experience. Sample sizes ranged from 4 to 317. Clinicians worked with ED patients with a variety of presentations and backgrounds, across a range of healthcare settings. Clinicians were aged 18 to 83 and were mostly White Females, with between 0.5 and 34+ years of experience working in this field. Not all studies specified whether clinicians worked in adult or child/adolescent services or inpatient/daypatient/outpatient/community services. See Figure 2 and Supplement Table 1.

### 3.1 Covid‐19 Pandemic

Four studies explicitly investigated clinicians’ experiences and service provision during the Covid‐19 pandemic(Colleluori et al. 2021; Lloyd et al. 2022; Novack et al. 2023; Shaw et al. 2021). Clinicians perceived a diminished therapeutic alliance due to having to deliver treatment via telemedicine, reporting feelings of therapeutic inefficacy and impaired communication due to the more limited non‐verbal communication in remote encounters and the use of personal protective equipment during face‐to‐face meetings (Colleluori et al. 2021). Increased emotional distress was highly prevalent due to isolation and infection anxiety (Colleluori et al. 2021; Lloyd et al. 2022). Evidently, the increased numbers and severity of ED presentations to services, coupled with a lack of resources and staff, were major contributors to clinician distress (Novack et al. 2023; Shaw et al. 2021). With time, clinicians began accepting the vast changes brought by the pandemic. Virtual appointments were praised for their increased flexibility and accessibility, though were not rated as appropriate for high‐risk patients.

### 3.2 Patient Complexity

Numerous studies named the challenges of working with ED patients and how to appropriately deliver care, including difficulties around treatment ambiguity, working with comorbidities, and patient risk. Clinicians might have simultaneously had feelings of uncertainty and lack of preparedness, especially as guidelines and treatments evolve (Levas‐Luckman 2014; Webb et al. 2022). Several clinicians reported not feeling skilled enough to support patients and mentioned the difficulties surrounding comorbidities and complex presentations (Bommen et al. 2023). McMaster et al. (2022) outlined how clinicians approached complexities around nutrition and collaboration with dietitians, concluding that clinicians needed further education on malnutrition, regardless of patient weight status or diagnosis, and the need to collaborate with dietitians. Ultimately, multilevel multidisciplinary collaboration was encouraged due to complexities of care (Chang et al. 2023; Corral‐Liria et al. 2022; Lachal et al. 2023; Webb et al. 2022).

Some patients may require nasogastric (NG) feeding and/or mealtime support in their treatment – five papers discussed clinicians’ experiences with these events in ED care (Cowan 2020; Hage et al. 2017; Kodua et al. 2020; Matthews‐Rensch et al. 2023; Watt and Dickens 2018). Nurses administering NG feeds, especially when combined with manual restraint of the patient, might feel extremely distressed, though the protocols themselves were mostly tolerable if evidence‐based and deemed appropriate for the patient. Nurses reported that their first few experiences with NG feeds were extremely uncomfortable, though their distress decreased with time due to increased feelings of confidence and competence with NG feed administration. Cowan (2020) found that overall stigmatization of ED patients was low in clinicians, and there was no association between stigma levels and the use of involuntary NG treatment. Concerns about how to manage concurrent oral feeding during NG treatment were also reported – patients re‐establishing oral feeding required strong support from clinicians, necessitating consistent clinician availability. The importance of good coping strategies and strong support for nurses administering NG feeds were stressed. Watt and Dickens (2018) found that clinicians’ own emotion regulation whilst providing mealtime support was essential, and that there may be a need for more formal training in this area. Findings highlighted the difficulties experienced by clinicians during NG feeds and mealtimes. However, these challenges ameliorated as clinician confidence and competence increased. Clinicians benefited from support and communication, further training on NG feeds and mealtime support were encouraged. Clinical supervision was an important space for clinicians to share any such difficult feelings and experiences.

Few studies addressed clinicians’ experience of ED patients with comorbidities. Two studies focused on type 1 diabetes with disordered eating (T1DE) (Macdonald et al. 2018; Zaremba et al. 2019) and one on autism and ED (Kinnaird et al. 2017). Themes pertaining to clinicians’ experiences with T1DE included: clinician‐specific factors (e.g., feeling lack of knowledge and competence, emotional burden,), patient‐specific factors (e.g., patient engagement challenges), and healthcare system‐related challenges (e.g., staff shortage). Kinnaird et al.'s (2017) evaluation of clinicians working with comorbid anorexia nervosa and autism found poor confidence in treating the comorbidity. All three studies stressed the need for multidisciplinary collaboration, training/supervision, and specific pathways for treatment.

### 3.3 Job Satisfaction

Overall, whilst ED clinicians were tasked with intense work demands, job satisfaction was high across clinician groups. However, medical doctors and clinical psychologists had greater job satisfaction than nursing staff (Hage and Rø 2020). A study of occupational therapists found that job satisfaction was rated highly (mean 7.4/10), feeling rewarded by their work (Devery et al. 2018). Another study found high job satisfaction in relation to patient care, but poor job satisfaction concerning organizational factors (Davey et al. 2014). Clinicians have identified both formal and informal social support in the workplace as highly important for job satisfaction. Overwhelmingly, concerns over resources were common underpinnings of clinicians’ negative attitudes towards their job satisfaction (Webb et al. 2022).

### 3.4 Burnout and Emotional Exhaustion

Burnout, emotional exhaustion, and compassion fatigue were common concepts investigated in clinician wellbeing studies. In one study, compassion fatigue was experienced by 22% of clinicians, with most participants experiencing low or average levels of compassion fatigue (Retkiewicz 2022). Workload demands and job insecurity were predictive factors for compassion fatigue, whilst job satisfaction was associated with (though did not predict) a reduction in compassion fatigue. Less experienced ED clinicians tended to experience higher rates of compassion fatigue (Hamama‐Raz and Mazor 2023). This might have been related to the acquisition of coping skills, as more experienced clinicians have had more opportunities to develop their coping skills.

A qualitative study investigating compassion fatigue amongst dietitians found that the exposure to ED patients’ distress caused emotional exhaustion and numbness, and that being poorly prepared for these exposures would increase the risk of compassion fatigue (Honig 2019). Seeking support, setting boundaries, and self‐care might help clinicians to manage and prevent compassion fatigue, with greater awareness and education on compassion fatigue needed. In relation to emotional exhaustion, multiple studies (Davén et al. 2022; Levas‐Luckman 2014) describe the ‘emotional rollercoaster’ experienced by clinicians. Studies revealed clinicians’ feelings of emotional overwhelm whilst also seeking the strength to cope with work‐related emotional exhaustion. Bommen et al. (2023) found that clinicians sometimes felt like a ‘punisher’ rather than a ‘helper,’ as their clinical intervention was causing high levels of distress in patients. Many clinicians also had experienced severely emotionally impactful events such as violence, aggression, and suicide attempts from patients in inpatient settings. Frontline clinicians expressed feeling a lack of acknowledgement by more senior staff or management, voicing that their emotionally challenging work carried out over long hours was not appropriately remunerated.

Whilst compassion fatigue and emotional exhaustion were quite commonly observed, burnout was low amongst clinicians, regardless of age or gender (Hage et al. 2021). However, certain ED‐specific factors were associated with higher rates of burnout. Devery and colleagues (Devery et al. 2018) investigated factors associated with burnout in occupational therapists in ED care, finding strong correlations between patient challenges (e.g., high risk of relapse) and exhaustion.

One's cultural identity was also important to consider, with some clinicians of color expressing feelings of isolation and emotional exhaustion resulting from a lack of diversity in their field (Biang et al. 2024). Particularly, exhaustion stemmed from having to advocate for other clinicians and patients of color, needing to educate others, and from not seeing themselves represented in the field.

### 3.5 Attitudes and Beliefs About ED

ED clinicians generally had positive attitudes towards their work. Witnessing a patient's recovery had a positive impact on clinicians, with some citing the experience of witnessing recovery as their motivation to work in this field (Levas‐Luckman 2014), and referencing feelings of personal growth. Clinicians also mentioned the enjoyment of the relational aspects of their work, for example, developing a positive relationship with a patient during a long inpatient stay (Bommen et al. 2023). When facing difficulties in a patient's treatment, clinicians attempted to cope by holding a strong sense of hope for the patient – a facet of ED treatment that lived experience patients and carers said was pertinent in recovery. Conversely, Jing et al. (2024) found that most clinicians generally felt a prognostic pessimism about ED patients, though likely these negative attitudes originated from a lower confidence in ED assessment and treatment, which potentially hampered patient engagement and outcomes. A different study found that some early career clinicians experienced tension, anxiety and frustration, and feelings of inadequacy in their work (Wu and Chen 2021).

One study suggested that clinicians’ personal biases might hinder treatment (Tragantzopoulou and Giannouli 2023). Less experienced therapists might endorse certain biases such as how some patient groups might be inherently very difficult to treat (typically, this bias is about AN). Similarly, clinicians commonly experience self‐doubt– feelings of pressure and of vulnerability in their position (Tragantzopoulou and Giannouli 2023; Zugai et al. 2019), and a lack of confidence in their abilities. An investigation of Scandinavian clinicians found that the majority of clinicians in the study felt the least confident working with binge eating disorder, and viewed patients as being more responsible for their illness than patients with anorexia nervosa, for instance (Reas et al. 2021).

Two studies (Turner et al. 2014; D'Souza Walsh et al. 2019) addressed the association between clinicians’ own anxiety or worry and attitudes towards cognitive behavioral therapy (CBT) for ED. Walsh et al. found that clinician anxiety levels appeared to influence their beliefs in the effectiveness and outcomes of CBT. Similarly, Turner et al. observed a link between higher levels of clinician anxiety and worry about certain facets of CBT. As such, this anxiety or worry might influence how clinicians approach working with ED patients, thus this is a relevant consideration when providing training and supervision. It is suggested that the integration of lived experience perspectives into all aspects of service design and delivery could ameliorate these negative attitudes by fostering compassion and understanding (Jing et al. 2024; Wu and Chen 2021).

### 3.6 Alliance and Countertransference

Establishing a strong therapeutic alliance between patients and their clinicians is essential for the success of therapy, as it builds trust, engagement, and commitment(Graves et al. 2017). Some clinicians form close relationships with their patients (Levy 2013), though may find it difficult to set boundaries or present themselves with authority (Harken et al. 2017; Zugai et al. 2019). Studies also emphasize the importance of openly communicating with patients to develop and maintain a strong therapeutic alliance (Chang et al. 2023; Corral‐Liria et al. 2022; Tragantzopoulou and Giannouli 2023). Chang et al. (2023) describe how building trusting relationships with the patient and their carers at the beginning of therapy is essential. The therapeutic alliance takes time to develop and relies on open, honest and unhurried communication. Tight time restrictions on treatment length, may make this more difficult to achieve. Conversely, other clinicians report struggling to develop relationships with their patients due to patient difficulties (Zugai et al. 2018, 2019). One study found that the use of cognitive‐behavioral techniques including psychoeducation might strengthen therapist ratings of the therapeutic alliance (Giannopoulos and Hilsenroth 2024).

The psychodynamic concept of ‘countertransference’, defined as a clinician's emotional reactions towards their patients (‘transferring’ their feelings onto the patient) is helpful to consider in the context of therapy. For example, clinicians’ views of their patient's treatment motivation were strongly correlated with the strength of the therapeutic alliance (Wu and Chen 2021), and mediated by their countertransference patterns (Lev Ari et al. 2023). Specifically, if the clinician perceived the patient as having a strong motivation to change, they felt less hostile and helpless, and more positive towards the patient, which ultimately affected the therapeutic alliance. (Groth et al. 2020) found that clinicians experienced greater overinvolved countertransference when a patient had more trauma severity, pointing to the need to reflect on the therapeutic alliance and ensure boundaries are effectively set.

The impact of ED work on clinician body image and body satisfaction has occasionally been assessed, with one study reporting that therapeutic relationships might affect clinicians’ own body satisfaction, though this depends on clinicians’ pre‐existing body satisfaction and internalization of thin ideals (Levy 2013). Another study (Biang et al. 2024) found that some clinicians noticed a vulnerability from the exposure to patients’ ED thoughts. However, most clinicians tend to have positive attitudes towards their body, stating that ED work had positive effects on their body satisfaction (Levas‐Luckman 2014). No studies investigated clinicians’ own BMI in relation to their work as an ED clinician.

### 3.7 Lived Experience

Several studies included clinicians with lived experience of ED, focusing on how clinicians’ personal experiences might impact their practice, as well as clinicians’ views towards, and experiences of self‐disclosure to colleagues, supervisors, or patients. Personal ED histories often motivate clinicians to enter the field of EDs, with this experience enhancing empathy towards one's patients and providing insight and hope for the journey of treatment and recovery (de Vos et al. 2016; Welch 2023). Working with ED patients after experiencing an ED might also reinforce clinicians’ own recovery (Curry and Andriopoulou 2023; Welch 2023). However, some boundary issues arise with self‐projection onto one's patient or becoming over‐involved, as well as the need to accurately self‐evaluate one's own wellness (Williams and Haverkamp 2015). (King and Russon 2023) delineate processes underlying the use and management of lived experience in one's clinical work. The application of lived experience to clinical work, using a ‘checks and balances’ system of self‐reflection and monitoring helps to ensure that lived experience is used beneficially. ED history provides clinicians with several insights into the patient experience (Curry and Andriopoulou 2023). Particularly, lived experience clinicians understand the barriers of accessing treatment, and the feeling of being dismissed or not listened to by some healthcare providers. Lived experience clinicians also highlight the displacement of responsibility within the service – other clinicians not wanting to take responsibility for medically complex or risky patients – thus feeling the need to assume the caring responsibilities themselves (Curry and Andriopoulou 2023). The impact of this displacement of responsibility is unknown.

Self‐disclosure, i.e., mentioning one's personal history, is often done with considerable forethought and with the anticipation of stigmatization from others. Nonetheless, clinicians who do self‐disclose to their supervisors do not tend to regret their decision (Welch 2023), and often receive positive responses from patients. To best support lived experience clinicians and to ensure the beneficial application of personal histories to clinical work, there is a need for a clear definition of recovery, guidelines surrounding self‐disclosure, and considerable support from supervisors. Further work is needed on decision models and guidance assisting clinicians’ ED disclosure, as well as the development of safe environments for discussion around personal histories.

### 3.8 Transitions

Three studies investigated clinicians’ attitudes towards and perspectives on transitions in ED care. ED treatment may involve different transitions, such as age‐related service transitions (e.g., from a child and adolescent service to an adult service), service transitions across geographical settings (e.g., to university, to a new city), or transitions across levels of care (e.g., from inpatient to outpatient treatment). Studies identified in this review discuss clinicians’ views on university and age‐related transitions.

Clinicians viewed ED transitions as a challenging event, for both themselves, and for their patients (Stocker et al. 2022). For geographical transitions, such as for a move to university, barriers might be experienced due to variations across the health system. For instance, differences in service awareness of ED transition needs, flexibility, or resources might hinder the patient's transition process (Webb and Schmidt 2021). Clinicians often cite poor links and communications with universities as a challenge to supporting their patients, as well as the difficulty of supporting the patient in their new environment (for instance, in dealing with heightened social and academic pressures). Age‐related service transitions are viewed as complex and anxiety‐provoking. Clinicians reported a lack of confidence in ensuring a continuity of care (e.g., medical monitoring; (Coelho et al. 2024), and a lack of trust and collaboration between services. There are also gaps in how clinicians perceive patient autonomy and the role of carers in the transition process, potentially resulting in conflicts on how to best approach the transition. For instance, child and adolescent services might emphasize family involvement more than adult services. Suggestions point to the need for wider education for clinicians working in university settings, university staff and general practitioners to recognize EDs, as well as the challenging nature of transitions. Another suggestion is to shift the focus from age‐based care to needs‐based care, e.g., to focus less on distinct age categories, and rather adjust for the patient on a case‐by‐case basis. Following (Lennips et al. 2024), a modular approach to evaluating continuity of care (e.g., breaking down healthcare services into their respective modules and components) might reveal gaps and overlaps in ED care provision.

Working collaboratively with patients and providing treatments that support patient autonomy is important to consider at all stages of ED care. One study (Geller et al. 2023) found that clinician compassion was related to the use of collaborative support behaviors – suggesting an emphasis on a compassionate clinical environment to promote collaborative care. A further study reports that clinicians’ own energy levels influence their patience and openness with patients, creating a barrier for autonomy supportive treatment (Oliverio et al. 2022). Autonomy and collaboration are essential to encourage positive change and engagement in treatment, accentuating the need to recognize the factors influencing clinicians’ use of collaborative care, as well as encouraging clinicians to enhance their skills in autonomy‐supportive treatment.

### 3.9 Gender Competency

Gender competency in ED clinicians was investigated in three studies (Ferrucci et al. 2024; Ferrucci et al. 2023; Kinnaird et al. 2018). EDs usually present in cis‐gendered females, with less research knowledge of the treatment needs of men, gender‐diverse and transgender patients. Clinicians are aware of the male‐specific features of EDs that need to be considered, though most knowledge of gender is reported to come from self‐education. Treatment approaches generally do not differ for men, although it is important to accommodate gender diversity in terms of spaces and materials provided to patients, as this has been a predominantly female‐dominated service and illness. Suggestions included creating a standardized training curriculum on gender competency and the evaluation of such, including the impact on clinician competency and on patient outcomes/satisfaction, and emphasizing the importance of family support in treatment to patients and families. However, there was no consensus on which gender‐specific adaptations to make in treatment. Patient and public involvement (PPI) in this area will be critical for creating effective clinical guidance.

### 3.10 Resources and Accessibility

Resource and access issues have been identified, concerning services, clinicians and patients themselves. Food insecurity (FI) is a problem often overlooked by clinicians, though becoming increasingly relevant with the rises in costs of living. In (Macdonald et al. 2018; Zaremba et al. 2019) clinicians reported having limited knowledge of food insecurity in ED treatment and a lack of resources on how to address this. Recommendations were made for treatment guidance, training and resource development for FI in ED. Further, patient reach is a concern, with travel being difficult for some (Crest et al. 2024; Kuehne et al. 2023; Love 2018) due to cost or rural location.

A 2018 study of clinicians in Ireland (McNicholas et al. 2018) voiced mostly negative comments about the provision of services, with 63 out of 79 reporting negative appraisals, such as disappointment with poor accessibility of services, lack of specialist services, and poor geographical distribution of services. Coelho et al. (2024) similarly highlighted clinicians’ concerns regarding the lack of resources and geographical barriers to providing treatment in Canada. Clinicians requested more multidisciplinary teamwork and planning for less‐resourced areas.

It is also necessary to acknowledge family resources, such as some caregivers’ limited time to support their loved ones with their ED, e.g., the inability to take leave to attend family therapy sessions (Crest et al. 2024).

There is an increased emphasis on improving clinic accessibility and extending services to rural areas. (Mayer et al. 2024) found that clinicians showed generally positive attitudes towards the integration of digital interventions into routine care, with the potential to improve reach for those who may not be able to travel for in‐person assessments. Although digital interventions still miss individuals without access to technology or private spaces for therapy, they provide a small bridge for the big gap in treatment reach.

### 3.11 Risk of Bias

Most studies were judged as having a low risk of bias, with only three being low/moderate and three being moderate. These studies failed to fully describe their methods or procedure, which could impact the generalizability and reliability of these results. The risk of bias appraisal can be found in Supplement 2.

## 4 Discussion

This review identified three overarching themes in the literature on ED clinicians’ experiences and wellbeing: (1) emotional and cognitive responses to clinical work, (2) training and guidance needs, and (3) resource needs. The included studies were largely qualitative or mixed‐methods, with a focus on clinician narratives and perceptions. Evidence suggests that whilst ED clinicians demonstrate a deep sense of purpose and commitment to their work, they are often emotionally taxed due to patient complexity, lack of resources, and insufficient training, especially regarding newer diagnoses and comorbidities. These findings underline the need for systemic changes and educational support.

### 4.1 Clinician Emotional and Cognitive Responses

Clinician attitudes to their work are generally positive, though an ‘emotional rollercoaster’ is commonly experienced when working with EDs. This is reflected through clinicians’ dedication and recognition of the importance of their role, whilst experiencing some emotional toll from their work (Warren et al. 2013). Patient care sometimes induces strong emotions in the clinician, which can be distressing. Warren et al. (2013) documented worry and frustration arising from their work, as well as the high degree of patience and empathy needed to work in this field. EDs are frequently comorbid with self‐harm and/or suicidality(Cucchi et al. 2016), which may add to clinician worry and emotional exhaustion. Similarly, the high rates of trauma experienced by ED patients (Brewerton 2007) can also result in emotional exhaustion and compassion fatigue, or even secondary traumatic stress – defined as the stress incited by indirect exposure to another person's trauma (Bride 2007).

Studies postulate that workload demands and job insecurity are contributing factors influencing emotional exhaustion and compassion fatigue (Warren et al. 2012; Warren et al. 2013; Webb et al. 2022). Less experience may also contribute, since clinicians tend to acquire coping skills over time.

Whilst emotional exhaustion was the only subscale of burnout strongly experienced by clinicians, overall burnout levels were low (Hage et al. 2021). Warren et al. (2013) found significantly lower levels of cynicism and higher levels of personal accomplishment among ED clinicians compared to healthcare providers in other areas. This aligns with Levas‐Luckman. (2014) findings that ED clinicians often report personal growth and fulfillment in their work. Many clinicians hold hope for their patients’ recovery and maintain high interest in their work.

A notable theme was the influence of lived experience on clinicians’ motivation. Some clinicians reported personal ED history as a motivator to enter the field, which can enhance empathy and insight. However, ethical concerns arise regarding when someone is ‘recovered enough,’ appropriate self‐disclosure, and safeguarding practices(Bachner‐Melman et al. 2018). Additionally, clinicians’ body image may be impacted by ED work (Levy 2013), and caution is urged when discussing such issues among colleagues(Yim 2024). Recommendations include clearer guidance on self‐reflection, support mechanisms, and ethical frameworks for clinicians with lived experience.

Negative attitudes towards ED patients were uncommon but present, particularly regarding binge eating disorder (Reas et al. 2021) or the perceived difficulty of anorexia nervosa (AN) care. These attitudes may stem from limited experience, confidence, or education, and could be improved through targeted training. Further research is needed to examine the perspectives of clinicians in primary and secondary care settings, as they are often the first point of contact for ED patients. Additionally, multidisciplinary collaboration is crucial in ED treatment, so efforts to improve attitudes and knowledge should include a wide range of healthcare professionals.

### 4.2 Training and Guidance

This review highlighted several areas for improvement in clinician training and available guidance. Early‐career clinicians often reported less confidence and knowledge in ED treatment (Wu and Chen 2021), yet training should be an ongoing process for clinicians at all levels. Up‐to‐date guidance is critical, and unconscious biases or negative attitudes can be mitigated through education (Jing et al. 2024).

There is a particular need for expanded training on newer diagnoses, comorbidities, and gender competency. Conditions such as ARFID, ED with comorbid autism, or T1DE were not widely discussed in the reviewed studies, highlighting major gaps in knowledge. Similarly, the needs of gender minorities in ED services are under‐researched. Training on transitions, collaborative care, and continuity across services is also essential. While these initiatives may be resource‐intensive, they are necessary for improving care quality and clinician wellbeing.

Regular clinical supervision should be prioritized in service provision. Effective supervision has been associated with reduced burnout and may help clinicians process complex emotions and avoid compassion fatigue(Edwards et al. 2006). Clinicians should have safe, reflective spaces for peer and supervisor support.

Emerging programs such as the Eating Disorder Services for Adults training (Novogrudsky et al. 2024) and FREED clinician training on transitions(FREED from ED 2023) show promise in addressing some of these gaps. However, more research is needed to evaluate their long‐term impact on clinician wellbeing and service outcomes.

### 4.3 Resource Needs

Workload and limited service resources were found to impact job satisfaction, burnout, and training access. Heavy caseloads can prevent clinicians from engaging in professional development, further compounding stress and limiting the adoption of evidence‐based practices. Supervision may offer some space to process these experiences, though meaningful changes are likely to require organizational‐level acknowledgement and systemic support.

Recommendations for practice, research and policy include:

- Improved training provision and guidelines, especially for new diagnoses and diverse presentations.
- Enhanced multidisciplinary collaboration to manage complex cases.
- Expanded service availability, accessibility, and inclusivity.

In sum, this review highlights the duality in ED work: a high level of fulfillment paired with significant emotional demands. The evidence suggests that burnout is most experienced as emotional exhaustion, with compassion fatigue and secondary trauma emerging as notable risks. Clinician wellbeing is closely linked to training adequacy, supervision, and service resources. There is a pressing need for comprehensive, inclusive, and continuous training programs — particularly around comorbidities, gender diversity, and emerging diagnoses — as well as structural reforms that ensure adequate staffing and supervision. Further research should explore these findings in broader mental health contexts and across interdisciplinary teams.

### 4.4 Limitations

There are several limitations of this review. Firstly, studies lacked quantitative data and concrete measures of clinician wellbeing, which would have added to understandings of clinician wellbeing and training needs. Some studies have a mild/moderate risk of bias, mainly due to poor descriptions of methodology. Another consideration is that the studies were all conducted in a small set of high‐income countries, affecting the generalizability of the findings to other countries. Additionally, many of these studies failed to report important demographic information, including on age and ethnicity, further impacting the applicability of these findings to all ED clinicians. Future investigations should aim to address these limitations and further research in this area, especially to determine if these findings are specific to ED clinicians.

## 5 Conclusion

The present review reflects on the current experiences, needs, and wellbeing of ED clinicians. Job satisfaction tends to be high, notwithstanding organizational factors such as limited staffing or large workloads. Clinicians typically hold positive views and attitudes towards their work, barring the emotional fluctuations that may arise from ED care. Emotional toll appears to be derived from the nature of ED (e.g., chronic nature, distress) – especially when taking into consideration the rate of patient risk and comorbidity seen in ED. Less experience, knowledge or confidence might predispose clinicians to emotional exhaustion or negative attitudes towards patient groups (e.g., negative attitudes towards BED due to a lack of knowledge of the illness). Lived experience is seen as an asset for clinicians, enhancing their insight and hope for patient recovery, seeing that appropriate boundaries are held and supervision is provided. Research should also investigate whether the findings of this review are specific to clinicians in ED settings or if these extend to mental health clinicians in other areas. Multidisciplinary teamwork is essential in ED care, denoting the importance of improving attitudes and knowledge of a wide range of clinicians towards ED. Training expansion is needed for clinicians of all experience levels, especially in areas such as BED and other diagnoses, ED with comorbidities, and minority groups in ED treatment. Clearer guidelines need to be made for transitions, collaborative care, and continuity across services. Improved service availability, accessibility, and specialization are also encouraged. Overall, improvements in resourcing are essential for ED clinician wellbeing, and ultimately for patient care.

## Author Contributions

**Kat Novogrudsky:** conceptualization, data curation, formal analysis, investigation, methodology, original draft, review and editing. **Janet Treasure:** conceptualization, supervision, writing – review and editing. Øyvind Rø: writing – review and editing. **Ulrike Schmidt:** conceptualization, supervision, writing – review and editing.

## Ethics Statement

This scoping review did not require ethics approval, as it involved the synthesis of publicly available literature and did not involve direct interaction with human participants or the collection of new data.

## Consent

This scoping review involved only secondary data from existing literature and did not involve direct interaction with patients or participants.

## Conflicts of Interest

The authors declare no conflict of interest.

## Supporting information

supp table 1 Papers included in scoping review analysis.

supp table 2 study risk of bias appraisals.

## Data Availability

The data that support the findings of this study are available from the corresponding author upon reasonable request.

## References

[jclp70005-bib-0001] https://librarysearch.kcl.ac.uk/discovery/openurl?institution=44KCL_INST&vid=44KCL_INST:44KCL_INST&sid=OVID:ameddb&id=pmid:&id=doi:&genre=article&atitle=Factors+associated+with+professional+identity%2C+job+satisfaction+and+burnout+for+occupational+therapists+working+in+eating+disorders%3A+A+mixed+methods+study&title=Australian+Occupational+Therapy+Journal&issn=0045-0766&date=2018&volume=65&issue=6&spage=523&aulast=Devery+H&isbn=&__char_set=utf8.

[jclp70005-bib-0002] https://librarysearch.kcl.ac.uk/discovery/openurl?institution=44KCL_INST&vid=44KCL_INST:44KCL_INST&sid=OVID:ovftdb&id=pmid:&id=doi:10.1037%2Fint0000332&genre=article&atitle=Therapist+Ratings+of+Technique+and+Alliance+Among+Adults+With+Eating+Disorders%3A++Support+for+Integrative+Treatment.&title=Journal+of+Psychotherapy+Integration&issn=1053-0479&date=2024&volume=Publish+Ahead+of+Print&issue=4&spage=392&aulast=Giannopoulos%2C+Evangeline&isbn=&__char_set=utf8.

[jclp70005-bib-0003] https://librarysearch.kcl.ac.uk/discovery/openurl?institution=44KCL_INST&vid=44KCL_INST:44KCL_INST&sid=OVID:ovftdb&id=pmid:&id=doi:10.1037%2Fpro0000308&genre=article&atitle=Therapist+Factors+Related+to+the+Treatment+of+Adolescent+Eating+Disorders.&title=Professional+Psychology+-+Research+%26+Practice&issn=0735-7028&date=2020&volume=51&issue=5&spage=517&aulast=Groth%2C+Taylor&isbn=&__char_set=utf8.

[jclp70005-bib-0004] https://librarysearch.kcl.ac.uk/discovery/openurl?institution=44KCL_INST&vid=44KCL_INST:44KCL_INST&sid=OVID:psycdb&id=pmid:&id=doi:&genre=article&atitle=Body+satisfaction+among+direct+care+staff+members+working+in+residential+treatment+programs+for+eating+disorders.&title=Dissertation+Abstracts+International%3A+Section+B%3A+The+Sciences+and+Engineering&issn=0419-4217&date=2014&volume=75&issue=1-B%28E%29&spage=No&aulast=Levy%2C+Brianna+N&isbn=978-1-303-40475-7&__char_set=utf8.

[jclp70005-bib-0005] https://librarysearch.kcl.ac.uk/discovery/openurl?institution=44KCL_INST&vid=44KCL_INST:44KCL_INST&sid=OVID:psycdb&id=pmid:&id=doi:&genre=article&atitle=Clinicians%27+perceptions+regarding+treatment+of+individuals+with+eating+disorders+in+rural+communities.&title=Dissertation+Abstracts+International%3A+Section+B%3A+The+Sciences+and+Engineering&issn=0419-4217&date=2018&volume=78&issue=9-B%28E%29&spage=No&aulast=Love%2C+Logan+Gillen&isbn=978-1369721775&__char_set=utf8.

[jclp70005-bib-0006] https://librarysearch.kcl.ac.uk/discovery/openurl?institution=44KCL_INST&vid=44KCL_INST:44KCL_INST&sid=OVID:psycdb&id=pmid:&id=doi:&genre=article&atitle=Coercive+and+compulsive+treatment+of+eating+disorders%3A+Surveying+treatment+providers%3F+Attitudes+and+behavior.&title=Dissertation+Abstracts+International%3A+Section+B%3A+The+Sciences+and+Engineering&issn=0419-4217&date=2021&volume=82&issue=2-B&spage=No&aulast=Jessica%2C+Cowan&isbn=979-8662500037&__char_set=utf8.

[jclp70005-bib-0007] https://librarysearch.kcl.ac.uk/discovery/openurl?institution=44KCL_INST&vid=44KCL_INST:44KCL_INST&sid=OVID:psycdb&id=pmid:&id=doi:&genre=article&atitle=Compassion+fatigue+in+registered+dietitians+who+treat+patients+with+eating+disorders.&title=Dissertation+Abstracts+International%3A+Section+B%3A+The+Sciences+and+Engineering&issn=0419-4217&date=2020&volume=81&issue=7-B&spage=No&aulast=Honig%2C+Caryn+Alyce&isbn=978-1392385586&__char_set=utf8.

[jclp70005-bib-0008] https://librarysearch.kcl.ac.uk/discovery/openurl?institution=44KCL_INST&vid=44KCL_INST:44KCL_INST&sid=OVID:psycdb&id=pmid:&id=doi:&genre=article&atitle=Healthcare+professionals%27+capacity+for+compassion+and+interactions+with+people+diagnosed+with+eating+disorders.&title=Dissertation+Abstracts+International%3A+Section+B%3A+The+Sciences+and+Engineering&issn=0419-4217&date=2022&volume=83&issue=9-B&spage=No&aulast=Retkiewicz%2C+Emily&isbn=979-8780651758&__char_set=utf8.

[jclp70005-bib-0009] https://librarysearch.kcl.ac.uk/discovery/openurl?institution=44KCL_INST&vid=44KCL_INST:44KCL_INST&sid=OVID:psycdb&id=pmid:&id=doi:&genre=article&atitle=Recovered+clinicians+and+the+use+of+self-disclosure+in+the+treatment+of+eating+disorders.&title=Dissertation+Abstracts+International%3A+Section+B%3A+The+Sciences+and+Engineering&issn=0419-4217&date=2023&volume=84&issue=4-B&spage=No&aulast=Welch%2C+Cathryn+F&isbn=979-8351444109&__char_set=utf8.

[jclp70005-bib-0010] https://librarysearch.kcl.ac.uk/discovery/openurl?institution=44KCL_INST&vid=44KCL_INST:44KCL_INST&sid=OVID:psycdb&id=pmid:&id=doi:&genre=article&atitle=Working+with+medical+and+psychological+illness%3A+A+phenomenological+exploration+of+nurses%27+experiences+in+treating+eating+disorders.&title=Dissertation+Abstracts+International%3A+Section+B%3A+The+Sciences+and+Engineering&issn=0419-4217&date=2016&volume=76&issue=8-B%28E%29&spage=No&aulast=Levas-Luckman%2C+Diana&isbn=978-1321659207&__char_set=utf8.

[jclp70005-bib-0011] American Psychiatric Association . (2013). Diagnostic and Statistical Manual of Mental Disorders. (5th Edition ed.).

[jclp70005-bib-0012] Bachner‐Melman, R. , L. Lev‐Ari , A. H. Zohar , and S. L. Lev . 2018. “Can Recovery From an Eating Disorder be Measured? Toward a Standardized Questionnaire.” Frontiers in Psychology 9: 2456. 30618916 10.3389/fpsyg.2018.02456 PMC6297874

[jclp70005-bib-0013] Bommen, S. , H. Nicholls , and J. Billings . 2023. “Helper’ or ‘Punisher’? A Qualitative Study Exploring Staff Experiences of Treating Severe and Complex Eating Disorder Presentations in Inpatient Settings.” Journal of Eating Disorders 11, no. 1: 216. 10.1186/s40337-023-00938-1. 38062517 PMC10704651

[jclp70005-bib-0014] Brewerton, T. D. 2007. “Eating Disorders, Trauma, and Comorbidity: Focus on Ptsd.” Eating disorders 15, no. 4: 285–304. 17710567 10.1080/10640260701454311

[jclp70005-bib-0015] Bride, B. E. 2007. “Prevalence of Secondary Traumatic Stress Among Social Workers.” Social Work 52, no. 1: 63–70. 17388084 10.1093/sw/52.1.63

[jclp70005-bib-0016] Cardi, V. , N. Mallorqui‐Bague , G. Albano , A. M. Monteleone , F. Fernandez‐Aranda , and J. Treasure . 2018. “Social Difficulties as Risk and Maintaining Factors In Anorexia Nervosa: A Mixed‐Method Investigation.” Frontiers in Psychiatry 9: 12. 29535645 10.3389/fpsyt.2018.00012 PMC5834472

[jclp70005-bib-0017] Chang, Y. S. , F. T. Liao , L. C. Huang , and S. L. Chen . 2023. “The Treatment Experience of Anorexia Nervosa in Adolescents From Healthcare Professionals’ Perspective: A Qualitative Study.” International Journal of Environmental Research and Public Health 20, no. 1) (no pagination), Article: 794. 10.3390/ijerph20010794. 36613116 PMC9819642

[jclp70005-bib-0018] Coelho, J. S. , T. Pardiwala , S. K. Marshall , et al. 2024. “Clinical Care for Severe and Persistent Eating Disorders in Pediatric Populations: Perspectives of Health Professionals.” Journal of Eating Disorders 12, no. 1: 83. 10.1186/s40337-024-01044-6. 38886837 PMC11181587

[jclp70005-bib-0019] Colleluori, G. , I. Goria , C. Zillanti , S. Marucci , and L. Dalla Ragione . 2021. “Eating Disorders During COVID‐19 Pandemic: The Experience of Italian Healthcare Providers.” Eating and Weight Disorders ‐ Studies on Anorexia, Bulimia and Obesity 26, no. 8: 2787–2793. 10.1007/s40519-021-01116-5. PMC7871942 33560511

[jclp70005-bib-0020] Corral‐Liria, I. , M. Alonso‐Maza , J. González‐Luis , S. Fernández‐Pascual , R. Becerro‐de‐Bengoa‐Vallejo , and M. Losa‐Iglesias . 2022. “Holistic Nursing Care for People Diagnosed With an Eating Disorder: A Qualitative Study Based on Patients and Nursing Professionals' Experience.” Perspectives in Psychiatric Care 58, no. 2: 840–849. 10.1111/ppc.12858. 34031892

[jclp70005-bib-0021] Cowan, J. 2020. “Coercive and Compulsive Treatment of Eating Disorders: Surveying Treatment Providers? Attitudes and Behavior.” Dissertation Abstracts International: Section B: The Sciences and Engineering 82, no. 2–B. https://aura.antioch.edu/etds/568.

[jclp70005-bib-0022] Crest, P. , S. S. Vendlinski , R. Borges , J. Landsverk , and E. C. Accurso . 2024. “Interdisciplinary Perspectives on Accessing Specialty Evidence‐Based Treatment for Medicaid‐Insured Adolescents With Eating Disorders.” Journal of Eating Disorders 12, no. 1: 167. 10.1186/s40337-024-01124-7. 39438893 PMC11515759

[jclp70005-bib-0023] Cucchi, A. , D. Ryan , G. Konstantakopoulos , et al. 2016. “Lifetime Prevalence of Non‐Suicidal Self‐Injury in Patients With Eating Disorders: A Systematic Review and Meta‐Analysis.” Psychological Medicine 46, no. 7: 1345–1358. 26954514 10.1017/S0033291716000027

[jclp70005-bib-0024] Curry, E. E. , and P. Andriopoulou . 2023. “Dual‐Experiences” of Treatment for Anorexia Nervosa: An Interpretative Phenomenological Analysis of Experiences of Treatment by Service Providers Who Are Recovered Service Users.” Mental Health Review Journal 28, no. 4: 396–413. 10.1108/MHRJ-02-2022-0010.

[jclp70005-bib-0025] D'Souza Walsh, K. , L. Davies , H. Pluckwell , H. Huffinley , and G. Waller . 2019. “Alliance, Technique, Both, or More? Clinicians’ Views on What Works in Cognitive‐Behavioral Therapy for Eating Disorders.” International Journal of Eating Disorders 52, no. 3: 278–282. 10.1002/eat.23033. 30706955

[jclp70005-bib-0026] Davén, J. , O. Hellzen , and M. Häggström . 2022. “Encountering Patients With Anorexia Nervosa ‐ An Emotional Roller Coaster. Nurses' Lived Experiences of Encounters in Psychiatric Inpatient Care.” International Journal of Qualitative Studies on Health and Well‐Being 17, no. 1: 2069651. 10.1080/17482631.2022.2069651. 35481811 PMC9068011

[jclp70005-bib-0027] Davey, A. , J. Arcelus , and F. Munir . 2014. “Work Demands, Social Support, and Job Satisfaction in Eating Disorder Inpatient Settings: A Qualitative Study.” International Journal of Mental Health Nursing 23, no. 1: 60–68. 23413943 10.1111/inm.12014

[jclp70005-bib-0028] Devery, H. , J. N. Scanlan , and J. Ross . 2018. “Factors Associated With Professional Identity, Job Satisfaction and Burnout for Occupational Therapists Working in Eating Disorders: A Mixed Methods Study.” Australian Occupational Therapy Journal 65, no. 6: 523–532. https://access.ovid.com/custom/redirector/wayfless.html?idp=https://kclidpdev.kcl.ac.uk/idp/shibboleth&url=http://ovidsp.ovid.com/ovidweb.cgi?T=JS&CSC=Y&NEWS=N&PAGE=fulltext&D=amed&AN=6004382. 30019456 10.1111/1440-1630.12503

[jclp70005-bib-0029] Edwards, D. , P. Burnard , B. Hannigan , et al. 2006. “Clinical Supervision and Burnout: The Influence of Clinical Supervision for Community Mental Health Nurses.” Journal of Clinical Nursing 15, no. 8: 1007–1015. 16879545 10.1111/j.1365-2702.2006.01370.x

[jclp70005-bib-0030] Ferrucci, K. A. , K. L. Lapane , B. M. Jesdale , E. McPhillips , and C. E. Dubé . 2024. “Eating Disorder Specialist Views on Gender Competency and Education for Treating Gender Minority Patients.” The Journal of Behavioral Health Services & Research 51, no. 2: 232–249. 10.1007/s11414-023-09864-1. 37845583

[jclp70005-bib-0031] Ferrucci, K. A. , E. McPhillips , K. L. Lapane , B. M. Jesdale , and C. E. Dubé . 2023. “Provider Perceptions of Barriers and Facilitators to Care in Eating Disorder Treatment for Transgender and Gender Diverse Patients: A Qualitative Study.” Journal of Eating Disorders 11, no. 1: 36. 10.1186/s40337-023-00760-9. 36890569 PMC9993680

[jclp70005-bib-0032] FREED From ED . (2023). Eating Disorders Service Transitions. https://freedfromed.co.uk/eating-disorders-service-transitions.

[jclp70005-bib-0033] Garcia, C. , L. C. Abreu , J. L. S. Ramos , et al. 2019. “Influence of Burnout on Patient Safety: Systematic Review and Meta‐Analysis.” Medicina 55, no. 9: 553. 31480365 10.3390/medicina55090553 PMC6780563

[jclp70005-bib-0034] Geller, J. , A. Fernandes , A. C. Kelly , L. Samson , and S. Srikameswaran . 2023. “Collaborative Care in Eating Disorders Treatment: Exploring the Role of Clinician Distress, Self‐Compassion, and Compassion for Others.” Journal of Eating Disorders 11, no. 1: 57. 10.1186/s40337-023-00741-y. 37024928 PMC10080953

[jclp70005-bib-0035] Giannopoulos, E. , and M. Hilsenroth . 2024. “Therapist Ratings of Technique and Alliance Among Adults With Eating Disorders: Support for Integrative Treatment.” Journal of Psychotherapy Integration 34: 392–400. https://access.ovid.com/custom/redirector/wayfless.html?idp=https://kclidpdev.kcl.ac.uk/idp/shibboleth&url=http://ovidsp.ovid.com/ovidweb.cgi?T=JS&CSC=Y&NEWS=N&PAGE=fulltext&D=paovftz4&AN=00012244-202412000-00003.

[jclp70005-bib-0036] Graham, M. R. , S. Tierney , A. Chisholm , and J. R. E. Fox . 2020. “The Lived Experience of Working With People With Eating Disorders: A Meta‐Ethnography.” International Journal of Eating Disorders 53, no. 3: 422–441. 31904870 10.1002/eat.23215

[jclp70005-bib-0037] Graves, T. A. , N. Tabri , H. Thompson‐Brenner , et al. 2017. “A Meta‐Analysis of the Relation Between Therapeutic Alliance and Treatment Outcome In Eating Disorders.” International Journal of Eating Disorders 50, no. 4: 323–340. 28152196 10.1002/eat.22672

[jclp70005-bib-0038] Groth, T. , M. J. Hilsenroth , J. Gold , D. Boccio , and G. A. Tasca . 2020. “Therapist Factors Related to the Treatment of Adolescent Eating Disorders.” Professional Psychology ‐ Research & Practice 51, no. 5: 517–526. https://access.ovid.com/custom/redirector/wayfless.html?idp=https://kclidpdev.kcl.ac.uk/idp/shibboleth&url=http://ovidsp.ovid.com/ovidweb.cgi?T=JS&CSC=Y&NEWS=N&PAGE=fulltext&D=paovftw&AN=00001326-202010000-00010.

[jclp70005-bib-0039] Hage, T. W. , K. Isaksson Rø , and Ø. Rø . 2021. “Burnout Among Staff on Specialized Eating Disorder Units In Norway.” Journal of Eating Disorders 9: 138. 34706769 10.1186/s40337-021-00473-x PMC8555148

[jclp70005-bib-0040] Hage, T. W. , and Ø. Rø . 2020. “Job Satisfaction At Specialized Eating Disorder Units In Norway.” International Journal of Eating Disorders 53, no. 12: 2044–2048. 10.1002/eat.23394. 33128294

[jclp70005-bib-0041] Hage, T. W. , Ø. Rø , and A. Moen . 2017. “Do You See What I Mean?” Staff Collaboration In Eating Disorder Units During Mealtimes.” BMC Nursing 16: 40. 10.1186/s12912-017-0233-3. 28736503 PMC5520364

[jclp70005-bib-0042] Hamama‐Raz, Y. , and S. Mazor . 2023. “Professional Quality of Life Among Professionals Working With People With Eating Disorders: The Interplay Between Meaning In Work, Optimism, and Career Duration.” Journal of Multidisciplinary Healthcare 16: 3249–3259. 10.2147/JMDH.S433458. 37936912 PMC10627083

[jclp70005-bib-0043] Harken, W. , J. Maxwell , M. Hainline , L. Pollack , and C. Roberts . 2017. “Perceptions of Caring for Adolescents With Eating Disorders Hospitalized on a General Pediatric Unit.” Journal of Pediatric Nursing 34: e34–e41. 10.1016/j.pedn.2017.02.008. 28283207

[jclp70005-bib-0044] Honig, C. A. 2019. “Compassion Fatigue In Registered Dietitians Who Treat Patients With Eating Disorders.” Doctoral diss., Walden University. https://www.proquest.com/docview/2343159618?pq-origsite=gscholar&fromopenview=true&sourcetype=Dissertations%20&%20Theses.

[jclp70005-bib-0045] Jing, E. , E. Gregertsen , L. Chen , and J. Russell . 2024. “Knowledge and Attitudes Towards Eating Disorders: A Survey of Psychiatrists and Psychiatry Trainees In New South Wales.” Australasian Psychiatry 32, no. 4: 375–382. 10.1177/10398562241249906. 38705873

[jclp70005-bib-0046] King, A. A. , and J. M. Russon . 2023. “Bringing and Removing Self From the Table”: Therapists’ Use and Management of Eating Disorder Lived Experience In the Treatment of Clients With Eating Disorders.” Journal of Marital and Family Therapy 49, no. 3: 654–674. 10.1111/jmft.12646. 37403806

[jclp70005-bib-0047] Kinnaird, E. , C. Norton , and K. Tchanturia . 2017. “Clinicians’ Views on Working With Anorexia Nervosa and Autism Spectrum Disorder Comorbidity: A Qualitative Study.” BMC Psychiatry 17, no. 1) (no pagination), Article: 292. 10.1186/s12888-017-1455-3. 28797223 PMC5553805

[jclp70005-bib-0048] Kinnaird, E. , C. Norton , and K. Tchanturia . 2018. “Clinicians’ Views on Treatment Adaptations for Men With Eating Disorders: A Qualitative Study.” BMJ Open 8, no. 8) (No Pagination), Article: e021934. 10.1136/bmjopen-2018-021934. PMC6078225 30082358

[jclp70005-bib-0049] Kodua, M. , J. M. Mackenzie , and N. Smyth . 2020. “Nursing Assistants’ Experiences of Administering Manual Restraint for Compulsory Nasogastric Feeding of Young Persons With Anorexia Nervosa.” International Journal of Mental Health Nursing 29, no. 6: 1181–1191. 10.1111/inm.12758. 32578949

[jclp70005-bib-0050] Kuehne, C. , A. Hemmings , M. Phillips , et al. 2023. “A UK‐Wide Survey of Healthcare Professionals’ Awareness, Knowledge and Skills of the Impact of Food Insecurity on Eating Disorder Treatment.” Eating behaviors 49 (No Pagination), Article: 101740. 10.1016/j.eatbeh.2023.101740. 37187140 PMC10775155

[jclp70005-bib-0051] Lachal, J. , E. Carretier , C. Prevost , et al. 2023. “The Experience of Healthcare Professionals Treating Adolescents With Eating Disorders In Psychiatric and Pediatric Inpatient Units for Adolescents: A Qualitative Study.” L'Encephale 49, no. 4: 331–341. 10.1016/j.encep.2022.01.015. 35725521

[jclp70005-bib-0052] Lennips, A. J. , V. J. T. Peters , B. R. Meijboom , A. C. Nissen , and J. E. H. Bunt . 2024. “Continuity of Care for Children With Anorexia Nervosa In the Netherlands: A Modular Perspective.” European Journal of Pediatrics 183: 2463–2476. 10.1007/s00431-024-05497-4. 38470519 PMC11035398

[jclp70005-bib-0053] Lev Ari, H. S. , M. Safyon , and R. Tuval‐Mashiach (2023). Clinician Reports of the Motivation to Change and the Therapeutic Alliance in Patients With Anorexia Nervosa: Countertransference as a Mediator [Empirical Study Quantitative Study]. *Journal of Psychotherapy Integration*, No Pagination Specified. 10.1037/int0000319.

[jclp70005-bib-0054] Levas‐Luckman, D. 2014. “Working With Medical and Psychological Illness: A Phenomenological Exploration of Nurses’ Experiences In Treating Eating Disorders.” Diss., University of Northern Colorado. https://digscholarship.unco.edu/dissertations/61.

[jclp70005-bib-0055] Levy, B. N. 2013. “Body Satisfaction Among Direct Care Staff Members Working In Residential Treatment Programs for Eating Disorders.” Doctoral Diss., University of Massachusetts. https://www.proquest.com/docview/1443846016?pq-origsite=gscholar&fromopenview=true&sourcetype=Dissertations%20&%20Theses.

[jclp70005-bib-0056] Lloyd, L. , D. Martin , S. Carney , M. Tattersall , and A. Basu . 2022. “Eating Disorders International Conference Abstracts 2022.” European Eating Disorders Review 30, no. 6: 830–845. 10.1002/erv.2916.

[jclp70005-bib-0057] Love, L. G. 2018. “Clinicians’ Perceptions Regarding Treatment of Individuals With Eating Disorders In Rural Communities [Dissertation Empirical Study; Interview; Nonclinical Case Study; Qualitative Study].” Dissertation Abstracts International: Section B: The Sciences and Engineering 78, no. 9–B(E: 1–24. https://www.proquest.com/docview/1893714801?pq-origsite=gscholar&fromopenview=true&sourcetype=Dissertations%20&%20Theses.

[jclp70005-bib-0058] Macdonald, P. , C. Kan , M. Stadler , et al. 2018. “Eating Disorders In People With Type 1 Diabetes: Experiential Perspectives of Both Clients and Healthcare Professionals.” Diabetic Medicine 35, no. 2: 223–231. 10.1111/dme.13555. 29178332

[jclp70005-bib-0059] Maslach, C. , and M. Leiter . 2006. *Burnout. Stress and Quality of Working Life* 37: 42–49.

[jclp70005-bib-0060] Maslach, C. , and M. P. Leiter . 2008. “Early Predictors of Job Burnout and Engagement.” Journal of Applied Psychology 93, no. 3: 498–512. 18457483 10.1037/0021-9010.93.3.498

[jclp70005-bib-0061] Matthews‐Rensch, K. , A. Young , C. Cutmore , A. Davis , S. Jeffrey , and S. Patterson . 2023. “Acceptability of Using a Nasogastric Refeeding Protocol With Adult Patients With Medically Unstable Eating Disorders.” Journal of Evaluation in Clinical Practice 29, no. 1: 49–58. 10.1111/jep.13718. 35700213

[jclp70005-bib-0062] Mayer, G. , D. Lemmer , I. Michelsen , P. Schrader , H. C. Friederich , and S. Bauer . 2024. “Views of German Mental Health Professionals on the Use of Digital Mental Health Interventions for Eating Disorders: A Qualitative Interview Study.” Journal of Eating Disorders 12, no. 1: 32. 10.1186/s40337-024-00978-1. 38395950 PMC10885453

[jclp70005-bib-0063] McNicholas, F. , C. O'Connor , N. McNamara , and L. O'Hara . 2018. “Eating Disorder Services for Young People in Ireland: Perspectives of Service Providers, Service Users and the General Adolescent Population.” Irish Journal of Psychological Medicine 35, no. 4: 301–309. 10.1017/ipm.2015.66. 30501667

[jclp70005-bib-0064] Methley, A. M. , S. Campbell , C. Chew‐Graham , R. McNally , and S. Cheraghi‐Sohi . 2014. “Pico, Picos and Spider: A Comparison Study of Specificity and Sensitivity In Three Search Tools for Qualitative Systematic Reviews.” BMC Health Services Research 14, no. 1: 579. 25413154 10.1186/s12913-014-0579-0 PMC4310146

[jclp70005-bib-0065] Novack, K. , R. Dufour , L. Picard , et al. 2023. “Canadian Pediatric Eating Disorder Programs and Virtual Care During the COVID‐19 Pandemic: A Mixed‐Methods Approach to Understanding Clinicians’ Perspectives.” Annals of General Psychiatry 22, no. 1) (No Pagination): 16. Article 16. 10.1186/s12991-023-00443-4. 37101241 PMC10132795

[jclp70005-bib-0066] Novogrudsky, K. , T. Gray , E. Mitchell , et al. 2024. “A Novel Whole‐Team Training Programme for Adult Eating Disorder Services In England: Rationale, Development and Preliminary Evaluation.” BJPsych Bulletin 48: 1–9. 10.1192/bjb.2024.20 PMC12171845 38616710

[jclp70005-bib-0067] Oliverio, S. , H. Steiger , A. St‐Hilaire , et al. 2022. “Barriers and Facilitators to Providing Autonomy Supportive Counselling to Individuals Seeking Treatment for An Eating Disorder.” Eating and Weight Disorders ‐ Studies on Anorexia, Bulimia and Obesity 27, no. 7: 2919–2929. 10.1007/s40519-022-01395-6. 35366169

[jclp70005-bib-0068] Page, M. J. , J. E. McKenzie , and P. M. Bossuyt , et al. 2021. “The PRISMA 2020 Statement: An Updated Guideline for Reporting Systematic Reviews.” BMJ 29: 372. 10.1136/bmj.n71 PMC8005924 33782057

[jclp70005-bib-0069] Pluye, P. , M.‐P. Gagnon , F. Griffiths , and J. Johnson‐Lafleur . 2009. “A Scoring System for Appraising Mixed Methods Research, and Concomitantly Appraising Qualitative, Quantitative and Mixed Methods Primary Studies In Mixed Studies Reviews.” International Journal of Nursing Studies 46, no. 4: 529–546. 19233357 10.1016/j.ijnurstu.2009.01.009

[jclp70005-bib-0070] Reas, D. L. , R. Isomaa , K. Solhaug Gulliksen , and J. Levallius . 2021. “Clinicians as a Critical Link: Understanding Health Professionals’ Beliefs and Attitudes Toward Anorexia Nervosa, Bulimia Nervosa, and Binge Eating Disorder.” Scandinavian Journal of Psychology 62, no. 6: 775–779. 10.1111/sjop.12777. 34569633

[jclp70005-bib-0071] Retkiewicz, E. 2022. “Healthcare Professionals’ Capacity for Compassion and Interactions With People Diagnosed With Eating Disorders [Dissertation Literature Review; Systematic Review].” Dissertation Abstracts International: Section B: The Sciences and Engineering 83, no. 9–B: 1–24. https://www.proquest.com/docview/2637957637?pq-origsite=gscholar&fromopenview=true&sourcetype=Dissertations%20&%20Theses.

[jclp70005-bib-0072] Seah, X. Y. , X. C. Tham , N. R. Kamaruzaman , and P. Yobas . 2017. “Knowledge, Attitudes and Challenges of Healthcare Professionals Managing People With Eating Disorders: A Literature Review.” Archives of Psychiatric Nursing 31, no. 1: 125–136. 28104050 10.1016/j.apnu.2016.09.002

[jclp70005-bib-0073] Shaw, H. , S. Robertson , and N. Ranceva . 2021. “What Was the Impact of a Global Pandemic (COVID‐19) Lockdown Period on Experiences Within An Eating Disorder Service? A Service Evaluation of the Views of Patients, Parents/Carers and Staff.” Journal of Eating Disorders 9, no. 1: 14. 10.1186/s40337-021-00368-x. 33468242 PMC7814524

[jclp70005-bib-0074] Sorenson, C. , B. Bolick , K. Wright , and R. Hamilton . 2016. “Understanding Compassion Fatigue In Healthcare Providers: A Review of Current Literature.” Journal of Nursing Scholarship 48, no. 5: 456–465. 27351469 10.1111/jnu.12229

[jclp70005-bib-0075] Stocker, A. , L. Rosenthal , L. Mesquida , J. P. Raynaud , and A. Revet . 2022. “Adult and Child and Adolescent Psychiatrists’ Experiences of Transition In Anorexia Nervosa: A Qualitative Study.” Journal of Eating Disorders 10, no. 1: 92. 10.1186/s40337-022-00610-0. 35788243 PMC9252565

[jclp70005-bib-0076] Taquet, M. , J. R. Geddes , S. Luciano , and P. J. Harrison . 2022. “Incidence and Outcomes of Eating Disorders During the COVID‐19 Pandemic.” The British Journal of Psychiatry 220, no. 5: 262–264. 10.1192/bjp.2021.105 PMC7612698 35048812

[jclp70005-bib-0077] Thompson‐Brenner, H. , D. A. Satir , D. L. Franko , and D. B. Herzog . 2012. “Clinician Reactions to Patients With Eating Disorders: A Review of the Literature.” Psychiatric Services 63, no. 1: 73–78. 22227763 10.1176/appi.ps.201100050

[jclp70005-bib-0078] Tragantzopoulou, P. , and V. Giannouli . 2023. ““You Feel That You Are Stepping Into a Different World”: Vulnerability and Biases In the Treatment of Anorexia Nervosa [Empirical Study Interview Qualitative Study].” European Journal of Psychotherapy & Counselling 25, no. 4: 351–368. 10.1080/13642537.2023.2278088.

[jclp70005-bib-0079] Treasure, J. , A. Crane , R. McKnight , E. Buchanan , and M. Wolfe . 2011. “First Do No Harm: Iatrogenic Maintaining Factors In Anorexia Nervosa.” European Eating Disorders Review 19, no. 4: 296–302. 21714039 10.1002/erv.1056

[jclp70005-bib-0080] Turner, H. , M. Tatham , M. Lant , V. A. Mountford , and G. Waller . 2014. “Clinicians’ Concerns About Delivering Cognitive‐Behavioural Therapy for Eating Disorders.” Behaviour Research and Therapy 57, no. 1: 38–42. 10.1016/j.brat.2014.04.003. 24793719

[jclp70005-bib-0081] de Vos, J. A. , C. Netten , and G. Noordenbos . 2016. “Recovered Eating Disorder Therapists Using Their Experiential Knowledge In Therapy: A Qualitative Examination of the Therapists’ and the Patients’ View.” Eating disorders 24, no. 3: 207–223. 10.1080/10640266.2015.1090869. 26467023 PMC4873721

[jclp70005-bib-0082] Warren, C. S. , M. E. Crowley , R. Olivardia , and A. Schoen . 2008. “Treating Patients With Eating Disorders: An Examination of Treatment Providers’ Experiences.” Eating Disorders 17, no. 1: 27–45. 10.1080/10640260802570098 19105059

[jclp70005-bib-0083] Warren, C. S. , K. J. Schafer , M. E. Crowley , and R. Olivardia . 2012. “A Qualitative Analysis of Job Burnout In Eating Disorder Treatment Providers.” Eating Disorders 20, no. 3: 175–195. 22519896 10.1080/10640266.2012.668476

[jclp70005-bib-0084] Warren, C. S. , K. J. Schafer , M. E. J. Crowley , and R. Olivardia . 2013. “Demographic and Work‐Related Correlates of Job Burnout In Professional Eating Disorder Treatment Providers.” Psychotherapy 50, no. 4: 553–564. 23795947 10.1037/a0028783

[jclp70005-bib-0085] Watt, J. , and G. L. Dickens . 2018. “Community‐Based Mealtime Management for Adolescents With Anorexia Nervosa: A Qualitative Study of Clinicians’ Perspectives and Experiences.” Journal of Child and Adolescent Psychiatric Nursing 31, no. 1: 30–38. 10.1111/jcap.12206. 30160075

[jclp70005-bib-0086] Webb, H. , B. Dalton , M. Irish , et al. 2022. “Clinicians’ Perspectives on Supporting Individuals With Severe Anorexia Nervosa In Specialist Eating Disorder Intensive Treatment Settings.” Journal of Eating Disorders 10, no. 1: 1–13. 34991715 10.1186/s40337-021-00528-z PMC8733908

[jclp70005-bib-0087] Webb, H. , and U. Schmidt . 2021. “Facilitators and Barriers to Supporting Young People With Eating Disorders During Their Transition To, and Time At, University: An Exploration of Clinicians’ Perspectives.” European Eating Disorders Review 29, no. 3: 443–457. 10.1002/erv.2795. 33044033

[jclp70005-bib-0088] Welch, C. F. 2023. “Recovered Clinicians and the Use of Self‐Disclosure In the Treatment of Eating Disorders [Dissertation Empirical Study; Qualitative Study].” Dissertation Abstracts International: Section B: The Sciences and Engineering 84, no. 4–B: 1–24. https://www.proquest.com/docview/2720090210?pq-origsite=gscholar&fromopenview=true&sourcetype=Dissertations%20&%20Theses.

[jclp70005-bib-0089] Williams, M. , and B. E. Haverkamp . 2015. “Eating Disorder Therapists’ Personal Eating Disorder History and Professional Ethics: An Interpretive Description.” Eating disorders 23, no. 5: 393–410. 25719397 10.1080/10640266.2015.1013393

[jclp70005-bib-0090] Wu, J. , J. Liu , S. Li , H. Ma , and Y. Wang . 2020. “Trends In the Prevalence and Disability‐Adjusted Life Years of Eating Disorders From 1990 to 2017: Results From the Global Burden of Disease Study 2017.” Epidemiology and Psychiatric Sciences 29: e191. 33283690 10.1017/S2045796020001055 PMC7737181

[jclp70005-bib-0091] Wu, W. L. , and S. L. Chen . 2021. “Nurses’ Perceptions on and Experiences In Conflict Situations When Caring for Adolescents With Anorexia Nervosa: A Qualitative Study.” International Journal of Mental Health Nursing 30, no. Suppl 1: 1386–1394. 10.1111/inm.12886. 34047043

[jclp70005-bib-0092] Yim, S. H. 2024. “Clinician Bodies In Eating Disorder Services: A Commentary.” Eating Disorders 33: 409–417. 38845209 10.1080/10640266.2024.2358267

[jclp70005-bib-0093] Zaremba, N. , A. Watson , C. Kan , et al. 2019. “Multidisciplinary Healthcare Teams’ Challenges and Strategies In Supporting People With Type 1 Diabetes to Recover From Disordered Eating.” Diabetic Medicine 37: 1992–2000. 10.1111/dme.14207. 31833586

[jclp70005-bib-0094] Zugai, J. S. , J. Stein‐Parbury , and M. Roche . 2018. “Therapeutic Alliance, Anorexia Nervosa and the Inpatient Setting: A Mixed Methods Study.” Journal of Advanced Nursing 74, no. 2: 443–453. 10.1111/jan.13410. 28792604

[jclp70005-bib-0095] Zugai, J. S. , J. Stein‐Parbury , and M. Roche . 2019. “Dynamics of Nurses’ Authority In the Inpatient Care of Adolescent Consumers With Anorexia Nervosa: A Qualitative Study of Nursing Perspectives.” International Journal of Mental Health Nursing 28, no. 4: 940–949. 10.1111/inm.12595. 30931550

