# Supplementary material for: A Scoping Review of Eating Disorder Clinicians' Experiences, Needs, Views and Wellbeing
Source: J Clin Psychol. 2025 Jun 16;81(10):903–17. doi: 10.1002/jclp.70005 (PMC12419239; doi:10.1002/jclp.70005)
Supplement: Supplementary file 1 — supp table 1 Papers included in scoping review analysis. [file JCLP-81-903-s001.docx]

Supplementary table 1. Papers included in scoping review analysis.

Note: CYP = children and young people’s services. IP = inpatient services. OP = outpatient services. DP = day patient services. SEED = severe and enduring eating disorder.

| Authors | Paper | Date | Location | Setting | Patient Diagnosis | Study Design | Clinician Characteristics | Summary of Relevant Findings |
| --- | --- | --- | --- | --- | --- | --- | --- | --- |
| Biang, A., Merlin‐Knoblich, C., and Lim, J. H. | An exploration of counselors of color working in the eating disorder field. | 2024 | USA | CYP, Adult, IP, OP, Private | AN | Qualitative Interview | Clinicians of colour, some with lived experience | Highlights challenges such as lack of diversity in the field, navigating racism and cultural mismatch, and the need for culturally informed supervision and mentorship. |
| Bommen, Nicholls and Billings | Helper' or 'punisher'? A qualitative study exploring staff experiences of treating severe and complex eating disorder presentations in inpatient settings | 2023 | UK | IP, all age | SEED, often with comorbidities | Qualitative interview | MDT | Inpatient staff felt moral distress from delivering involuntary treatment, with limited organizational support but strong peer support. Coping involved hope and relational care. The study calls for better recruitment, retention, and understanding of trauma impacts. |
| Chang, Liao, Huang and S.Chen | The Treatment Experience of Anorexia Nervosa in Adolescents from Healthcare Professionals' Perspective: A Qualitative Study | 2023 | Taiwan | CYP | AN | Qualitative interview | MDT | Building trust with clients and parents, consistent team goals, and family involvement are key to successful treatment. Education, collaboration, and flexible interaction strategies support care for adolescents with anorexia. |
| Coelho, J. S., Pardiwala, T., Marshall, S. K., Lam, P. Y., Grewal, S., Virani, A., ... and Geller, J. | Clinical care for severe and persistent eating disorders in pediatric populations: Perspectives of health professionals. | 2024 | Canada/Australia | CYP, all age, IP, OP, Private |  | Mixed-methods survey | MDT | Clinicians reported limited resources, staff and caregiver burnout, and ethical concerns about labeling young people as having severe and enduring eating disorders. While some saw value in quality-of-life interventions, others questioned their suitability for pediatric populations. |
| Colleluori, Goria, Zillanti, Marucci and Dalla Ragione | Eating disorders during COVID-19 pandemic: the experience of Italian healthcare providers | 2021 | Italy |  | AN, BN, BED | Mixed-methods survey | MDT | Healthcare professionals reported feelings of therapeutic inefficacy, compromised therapeutic alliance, and difficulties engaging families during the pandemic. Challenges included limited non-verbal communication, use of PPE, and infection-related anxiety. Stress management strategies and further research were recommended. |
| Corral-Liria, Alonso-Maza, Gonzalez-Luis, Fernandez-Pascual, Becerro-de-Bengoa-Vallejo and Losa-Iglesias | Holistic nursing care for people diagnosed with an eating disorder: A qualitative study based on patients and nursing professionals' experience | 2022 | Spain | Adult |  | Qualitative interview | Nurses | Nurses play a key, often unanticipated role in recovery by offering holistic care beyond nutrition, addressing emotional, psychological, and social needs. Strong therapeutic alliances require open communication, teamwork, and coordinated efforts. More training and resources are needed to meet demand and enhance care quality. |
| Cowan | Coercive and compulsive treatment of eating disorders: Surveying treatment providers’ Attitudes and behavior | 2020 | USA | All |  | Quantitative survey | MDT | Treatment providers reported low stigma and low use of coercive methods, with no significant link between attitudes and the use of coercion or compulsion in care. |
| Crest, P., Vendlinski, S. S., Borges, R., Landsverk, J., & Accurso, E. C. | Interdisciplinary perspectives on accessing specialty evidence-based treatment for Medicaid-insured adolescents with eating disorders | 2024 | USA | CYP, IP, OP |  | Qualitative | MDT | Clinicians valued integrating mental health and medical care, especially in public systems. FBT training improved collaboration, patient access, and provider confidence. Barriers included weight stigma, referral issues, and lack of culturally competent care. Psychoeducation and culturally responsive services were seen as essential. |
| Curry and Andriopoulou | Dual-experiences of treatment for anorexia nervosa: An interpretative phenomenological analysis of experiences of treatment by service providers who are recovered service users | 2023 | UK |  | AN | Qualitative interview | Lived experience | Professionals with lived experience of AN described barriers like dismissive gatekeepers and avoidance of responsibility by clinicians. Lived experience was seen as enhancing empathy, understanding, and therapeutic relationships. Greater compassion and insight into AN among professionals were seen as crucial for improving care. |
| Daven, Hellzen and Haggstrom | Encountering patients with anorexia nervosa - An emotional roller coaster. nurses' lived experiences of encounters in psychiatric inpatient care | 2022 | Sweden | Adult IP | All | Qualitative interview | Nurses | Nurses experienced intense emotional challenges when caring for patients with anorexia, described as an "emotional rollercoaster." They coped by relying on colleagues, routines, and building emotional resilience. Support to manage these emotional demands is crucial. |
| Davey, Arcelus and Munir | Work demands, social support, and job satisfaction in eating disorder inpatient settings: a qualitative study | 2014 | UK | Adult IP | All | Qualitative interview |  | ED inpatient care is complex, with heavy therapeutic, physical, and organizational demands. Staff rely heavily on workplace social support. Effective teamwork, supervision, and reduced administrative burdens are essential to improve care and staff wellbeing. |
| de Vos, Netten and Noordenbos | Recovered eating disorder therapists using their experiential knowledge in therapy: A qualitative examination of the therapists' and the patients' view | 2016 | Netherlands | OP, IP | All | Qualitative questionnaire | Lived experience | LE can enhance therapeutic relationships and instill hope, but self-disclosure should be used carefully. LE is seen as a valuable therapeutic tool, prompting calls for guidelines, a clear recovery definition, and more research on its impact in ED treatment. |
| Devery, Scanlan and Ross | Factors associated with professional identity, job satisfaction and burnout for occupational therapists working in eating disorders: A mixed methods study | 2018 | Australia |  | All | Mixed-methods survey and interview | Occupational therapists | OT in ED settings face structural and role-related challenges, including being underutilized. Maintaining an occupation-focused approach, supervision, and work-life balance help manage stress. More research and support are needed to strengthen the OT role and evidence base in ED care. |
| Downey, A. E., Odette, M., Sanders, A. E., Kuykendall, M., Saunders, E., Nagata, J. M., ... and Garber, A. K. | What medical providers need to elevate outpatient care for adolescents and young adults with binge-eating disorder: A novel protocol. | 2024 | USA | all | all | Quantitative | MDT | Providers felt only somewhat confident screening for BED in youth, with most uncertain about treatment recommendations. Medication was commonly considered for binge episodes or comorbidities. Key educational needs included medication use, mental health treatment, weight management, and diagnostic criteria. |
| Ferrucci, McPhillips, Lapane, Jesdale and Dube | Provider perceptions of barriers and facilitators to care in eating disorder treatment for transgender and gender diverse patients: a qualitative study | 2023 | USA | all | all | Qualitative interview | MDT | Clinicians emphasize the importance of training on gender but often rely on self-education. There’s a need for standardized, comprehensive education in graduate programs and ongoing training. Future research should examine gaps in gender education and its impact on care for gender minority patients with eating disorders. |
| Ferrucci, Lapane, Jesdale, McPhillips and Dube | Eating Disorder Specialist Views on Gender Competency and Education for Treating Gender Minority Patients | 2024 | USA | all | all | Qualitative interview | MDT | Barriers to care for gender minority patients include stigma, financial issues, lack of gender-competent care, and discrimination in treatment. Clinician attitudes and knowledge play a key role. Improvements in provider education and understanding are needed, with future research recommended to address provider-driven barriers. |
| Geller, Fernandes, Kelly, Samson and Srikameswaran | Collaborative care in eating disorders treatment: exploring the role of clinician distress, self-compassion, and compassion for others | 2023 | Canada | all | all | Quantitative survey | MDT | Clinician compassion was strongly linked to collaborative care, unlike general distress. Fostering compassionate clinical environments may enhance collaborative treatment approaches. |
| Giannopoulos, E., and Hilsenroth, M. | Therapist Ratings of Technique and Alliance Among Adults With Eating Disorders: Support for Integrative Treatment | 2024 | USA/Canada | Adult |  | Quantitative survey | MDT | Therapist use of cognitive-behavioural and psychodynamic–interpersonal techniques—especially psychoeducation and exploring emotions—positively predicted stronger therapeutic alliances. In contrast, concurrent inpatient/day treatment negatively impacted alliance. Results underscore the value of specific integrative interventions for improving ED treatment outcomes in adults. |
| Groth, Hilsenroth, Gold, Boccio and Tasca | Therapist Factors Related to the Treatment of Adolescent Eating Disorders | 2020 | USA/Canada | all | all | Quantitative survey | MDT | Therapists showed more overinvolvement and used more PI techniques with trauma-affected ED adolescents, especially in bulimia and binge eating cases. Findings support therapist reflection and boundary setting in trauma-informed care. |
| Hage and Ro | Job satisfaction at specialized eating disorder units in Norway | 2020 | Norway | IP/DP, adults, adolescents |  | Quantitative survey | MDT | Job satisfaction was generally high but varied by role and shift. Doctors and psychologists reported higher satisfaction than nurses. Tailored strategies are needed to improve satisfaction across roles and shift types. |
| Hage, Ro and Ro | Burnout among staff on specialized eating disorder units in Norway | 2021 | Norway | IP/DP, adults, adolescents |  | Quantitative survey | MDT, some with LE | Burnout levels were low, but ED-specific factors and emotional dissonance predicted key burnout aspects. Treatment context and population are important to consider when examining burnout. |
| Hage, Ro and Moen | "Do you see what I mean?" staff collaboration in eating disorder units during mealtimes | 2017 | Norway | IP, adults | Mostly AN | Qualitative interview | Milieu therapists | Collaboration during mealtimes in ED units involves strategic seating, communication, and experience. Understanding these practices can improve staff training and patient support through clearer routines and targeted interventions. |
| Hamama-Raz and Mazor | Professional Quality of Life Among Professionals Working with People with Eating Disorders: The Interplay Between Meaning in Work, Optimism, and Career Duration | 2023 | Israel |  |  | Quantitative survey | MDT | Longer ED ward experience reduced secondary traumatic stress. Optimism and meaning in work increased compassion satisfaction and lowered burnout. The benefit of meaning in work on satisfaction decreased with higher optimism. Burnout risk was higher in staff with under 12.3 years’ experience. Regular reflection and supervision are recommended to support staff wellbeing. |
| Harken, Maxwell, Hainline, Pollack and Roberts | Perceptions of Caring for Adolescents With Eating Disorders Hospitalized on a General Pediatric Unit | 2017 | USA | Tertiary hospital, IP, CYP |  | Qualitative interview | MDT | System changes like centralized admission, standardized protocols, consult teams, and staff training improved ED care. With support, nurses found ED work rewarding despite initial challenges. Standardized tools and education help create a stronger therapeutic environment. |
| Honig | Compassion fatigue in registered dietitians who treat patients with eating disorders | 2019 | USA | All |  | Qualitative interview | Dietitians | Repeated exposure to pain and suffering led to emotional exhaustion and numbness. The risk of compassion fatigue is high when unprepared for trauma exposure. Seeking support, setting boundaries, self-care, and education on compassion fatigue are crucial. |
| Jing, E., Gregertsen, E., Chen, L., and Russell, J. | Knowledge and attitudes towards eating disorders: A survey of psychiatrists and psychiatry trainees in New South Wales | 2024 | Australia | All | AN, BN, BED | Mixed-methods | Psychiatrists | Knowledge gaps and pessimistic views of EDs lowered clinicians’ confidence and could impair engagement. Incorporating lived experience may boost understanding, compassion, and therapeutic alliance. |
| King and Russon | Bringing and Removing Self from the Table: Therapists' use and management of eating disorder lived experience in the treatment of clients with eating disorders | 2023 | USA | Mostly private practice |  | Qualitative interview | Lived experience therapists | Suggested that LE can be transformed into clinical guidance with appropriate boundaries and allowing for differences of experience to emerge. Personal factors influence clinicians’ approaches to applying LE into their practice (e.g., values, stigma). |
| Kinnaird, Norton and Tchanturia | Clinicians' views on working with anorexia nervosa and autism spectrum disorder comorbidity: A qualitative study | 2017 | UK | OP, adult | AN w/ autism | Qualitative interview |  | Many clinicians lack confidence in treating comorbid ASD with ED, and treatment adaptations are based on individual experiences rather than a systematic approach. Research is needed to establish treatment guidelines and provide standardized support through training and treatment pathways. |
| Kinnaird, Norton and Tchanturia | Clinicians' views on treatment adaptations for men with eating disorders: A qualitative study | 2018 | UK | DP, OP, adult | Men with ED | Qualitative interview |  | Clinicians noted male-specific issues, and that standard ED treatments can be adapted within a supportive, male-friendly environment. Consensus is lacking on specific adaptations (e.g., male-only groups or male clinicians). Clear guidelines and research on best practices and treatment environment are needed. |
| Kodua, Mackenzie and Smyth | Nursing assistants' experiences of administering manual restraint for compulsory nasogastric feeding of young persons with anorexia nervosa | 2020 | UK | IP, CYP | AN with NG feeding | Qualitative interview | Nursing assistants | Manual restraint during compulsory NG feeds in AN patients is highly distressing for nursing assistants, causing emotional and physical strain. Coping strategies included peer support and emotional detachment. Support needs include fair distribution of restraint duties, proper training, debriefing, and access to reflective or therapeutic support. |
| Kuehne, Hemmings, Phillips, Ince, Chounkaria, Ferraro, et al. | A UK-wide survey of healthcare professionals' awareness, knowledge and skills of the impact of food insecurity on eating disorder treatment | 2023 | UK | Mostly public health services |  | Mixed-methods survey | MDT | Over 20% of ED patients were perceived by HCPs to have experienced FI, and most expected this issue to grow. Despite this, there is limited knowledge and few resources addressing FI in ED care. There is a clear need for routine screening, practical guidance, and formal training. |
| Lachal, Carretier, Prevost, Nadeau, Taddeo, Fortin, et al. | The experience of healthcare professionals treating adolescents with eating disorders in psychiatric and pediatric inpatient units for adolescents: A qualitative study | 2023 | Canada/France | IP, CYP |  | Qualitative interview | MDT | ED treatment in adolescents requires a flexible, evolving, and collaborative approach due to the complex interplay of psychiatric, medical, and psychosocial issues. A multilevel model of collaboration—ranging from multidisciplinary to transdisciplinary—should be tailored to each patient’s needs. |
| Lennips, Peters, Meijboom, Nissen and Bunt | Continuity of care for children with anorexia nervosa in the Netherlands: a modular perspective | 2024 | Netherlands | OP, CYP | AN | Qualitative interview | MDT | Barriers to transitioning from CAMHS to AMHS for patients with AN include differing treatment cultures, mistrust between services, clinician confidence issues, and weak alliance-building in AMHS. Written procedures are recommended to support smoother, more predictable transitions. |
| Lev Ari, Safyon and Tuval-Mashiach | Clinician reports of the motivation to change and the therapeutic alliance in patients with anorexia nervosa: Countertransference as a mediator | 2023 | Israel |  | AN | Quantitative survey | MDT | Therapists’ countertransference patterns mediated the relationship between patient motivation to change and the therapeutic alliance (TA). Supporting therapists in managing countertransference may help strengthen TA through enhanced understanding of patient motivation. |
| Levas-Luckman | Working with medical and psychological illness: A phenomenological exploration of nurses' experiences in treating eating disorders | 2016 | USA | IP, adolescent and adult |  | Qualitative int | Nurses | Themes of initial attitudes, treatment conditions, and emotional awareness shaped nurses’ experiences. Emphasizing these can improve training, support, and team communication, while a strengths-based approach may enhance outcomes and reduce negativity. |
| Levy | Body satisfaction among direct care staff members working in residential treatment programs for eating disorders | 2014 | USA | IP |  | Qualitative int | Direct care staff members | Close staff-client relationships in residential ED settings may be shaped by staff roles, personal history, and client diagnoses. Staff body satisfaction was impacted—positively or negatively—depending on their prior body image and internalized thin ideals. |
| Lloyd, Martin, Carney, Tattersall and Basu | Themes of recovery from a child and adolescent eating disorder unit: Staff experiences (abstract) | 2022 | USA | CYP |  | Mixed methods |  | Staff observed increased ED symptoms and emotional distress linked to lockdowns, with limited visits and social isolation worsening patient outcomes. Adaptations were made, but many staff have come to see COVID-19-related changes as a lasting part of their roles. |
| Lockertsen, Nilsen, Holm, Ro, Burger and Rossberg | Mental health professionals' experiences transitioning patients with anorexia nervosa from child/adolescent to adult mental health services: a qualitative study | 2020 | Norway | CYP to Adult, IP, OP | AN | Qualitative interview | Clinicians with experience in transitions | Barriers to transition from CAMHS to AMHS include differing treatment cultures, mistrust between services, clinician confidence issues, and lack of alliance-building. Written transition procedures are recommended to improve continuity of care for patients with AN. |
| Love | Clinicians' perceptions regarding treatment of individuals with eating disorders in rural communities | 2018 | USA |  | All | Qualitative interview | MDT | Clinicians in rural areas report limited resources for ED treatment, emphasizing the need for team-based care and addressing travel barriers. Organizational and training improvements are needed to better support rural mental health services. |
| Macdonald, Kan, Stadler, De Bernier, Hadjimichalis, Le Coguic, et al. | Eating disorders in people with Type 1 diabetes: experiential perspectives of both clients and healthcare professionals | 2018 | UK |  | T1DE | Qualitative interview | MDT | Key themes include challenges in service provision and dual diagnosis care, reflections on recovery, and the lived experience of T1D and ED. Highlights the need for multidisciplinary collaboration, staff training, supervision, and ongoing monitoring. |
| Matthews-Rensch, Young, Cutmore, Davis, Jeffrey and Patterson | Acceptability of using a nasogastric refeeding protocol with adult patients with medically unstable eating disorders | 2023 | Australia |  | NG feed | Qualitative interview | MDT | NG feeding acceptability varies based on staff competence, confidence, and the balance between patient-centered care and stigma. There is a need for improved communication, specialized education, and consideration of oral intake in treatment. Further research into co-designed, personalized care models is necessary. |
| Mayer, Lemmer, Michelsen, Schrader, Friederich and Bauer | Views of German mental health professionals on the use of digital mental health interventions for eating disorders: a qualitative interview study | 2024 | Germany | IP |  | Qualitative interview | MDT | Digital interventions (DMHIs) are accessible low-threshold options for ED patients. A strong therapeutic alliance is crucial. Concerns remain for severe cases, such as AN or suicidality, and clinicians feel inadequately informed about recent developments. Targeted DMHIs for specific ED diagnoses and blended care approaches are recommended. |
| McMaster, Wade, Franklin, Waller and Hart | Impact of patient characteristics on clinicians' decisions to involve dietitians in eating disorder treatment | 2022 | Australia | All |  | Quantitative survey | Dietitians | Clinicians involve dietitians based on patient factors—ED type, weight status, comorbidities, and treatment progress—rather than their own beliefs. There’s a need for education on malnutrition risks across all ED presentations and on the dietitian’s role in care. |
| McNicholas, OConnor, McNamara and O'Hara | Eating disorder services for young people in Ireland: Perspectives of service providers, service users and the general adolescent population | 2018 | Ireland | CYP |  | Mixed-methods | MDT | 32% of clinicians have noticed a shift in presentations from female toward younger, male patients. Criticism surrounds the quality of ED services, long wait times for CAMHS appointments, and insufficient staffing/resources. While many are aware of voluntary organizations, there is a need for clearer guidance and standardized protocols for ED care. |
| Novack, Dufour, Picard, Taddeo, Nadeau, Katzman, et al. | Canadian pediatric eating disorder programs and virtual care during the COVID-19 pandemic: a mixed-methods approach to understanding clinicians’ perspectives | 2023 | Canada |  |  | Mixed methods | MDT | The majority of pediatric ED care moved online during the pandemic. Key challenges included responding to increased demand with limited resources, adapting to changes due to COVID-19, and managing uncertainty. Health professionals generally had positive views on virtual care, considering it feasible and useful. Training in virtual interventions is recommended. |
| Oliverio, Steiger, St-Hilaire, Paquin-Hodge, Leloup, Israel, et al. | Barriers and facilitators to providing autonomy supportive (AS) counselling to individuals seeking treatment for an eating disorder | 2024 | Canada |  | all | Qualitative interviews | MDT | Organizational support and patient motivation facilitated AS interventions, while passive/help-rejecting behaviors and heavy workloads hindered them. Programs to build therapists’ AS skills and evaluate intervention components are recommended. |
| Reas, Isomaa, Solhaug Gulliksen and Levallius | Clinicians as a critical link: Understanding health professionals' beliefs and attitudes toward anorexia nervosa, bulimia nervosa, and binge eating disorder | 2021 | Norway |  | AN, BN, BED | Quantitative survey | MDT | Healthcare professionals perceived AN as the most severe, followed by BN and BED, viewing them as psychological rather than medical. Treatment for BN was seen as more effective, while professionals showed less confidence and enjoyment working with BED. Training in managing BED was identified as necessary for Nordic healthcare professionals. |
| Retkiewicz | Healthcare professionals' capacity for compassion and interactions with people diagnosed with eating disorders | 2022 | UK | all | all | Quantitative survey | MDT | Around 22% of healthcare professionals (HCPs) experienced high compassion fatigue, while 17% reported low compassion satisfaction. Workload demands and job insecurity were key predictors of compassion fatigue. Reducing workplace stressors and addressing emotion regulation strategies are recommended. |
| Ryu, Hamilton and Tarrant | Early career mental health nurses' emotional experiences in specialist eating disorder units, Victoria, Australia | 2022 | Australia | IP |  | Qualitative interview | Nurses | Early career nurses experienced anxiety, frustration, and powerlessness. Over time, strong emotions—anger, inadequacy, projection—were reframed as tools for empathy. Clinical supervision and reflective spaces are recommended to build resilience and professional growth. |
| Shaw, Robertson and Ranceva | What was the impact of a global pandemic (COVID-19) lockdown period on experiences within an eating disorder service? A service evaluation of the views of patients, parents/carers and staff | 2021 | UK | CYP | AN, BN, BED | Mixed methods | MDT | COVID-19 increased pressure on UK eating disorder services, with more acute cases and elevated risks. Virtual appointments provided flexibility but altered the relational experience. Face-to-face appointments at the start of treatment, family involvement, and strong staff support are essential. MDT and adequate staff resources are necessary to manage risks. |
| Stocker, Rosenthal, Mesquida, Raynaud and Revet | Adult and child and adolescent psychiatrists' experiences of transition in anorexia nervosa: a qualitative study | 2022 | UK | CYP to adult | AN | Qualitative interviews | Psychiatrists (physicians) | Physicians experienced AN patient transitions as anxiety-provoking. Rigid age-based transitions conflict with patient needs and autonomy. Tailoring transition timing to individual readiness and supporting physicians in facilitating patient autonomy may improve transition experiences. |
| Tragantzopoulou and Giannouli | "You feel that you are stepping into a different world": Vulnerability and biases in the treatment of anorexia nervosa | 2023 | Greece | Private | AN | Qualitative interviews | Therapists | Therapists’ self-doubt, biases, and cultural influences hinder effective treatment, especially when compounded by mistrust and parental involvement. Novice therapists should engage in self-reflection and seek supervision to address these barriers and improve their practice. |
| Turner, Tatham, Lant, Mountford and Waller | Clinicians' concerns about delivering cognitive-behavioural therapy for eating disorders | 2014 | UK |  |  | Quantitative survey | MDT | Clinicians were most concerned about addressing body image and ending treatment, with less concern around psychoeducation. Concerns clustered into process, education, cognitive, and exposure-related factors. Experience reduced worry, while higher anxiety increased it. Supporting clinicians' tolerance of uncertainty may enhance CBT delivery. |
| Walsh, Davies, Pluckwell, Huffinley and Waller | Alliance, technique, both, or more? Clinicians' views on what works in cognitive-behavioral therapy for eating disorders | 2019 | UK/USA |  |  | Quantitative survey | MDT | Higher anxiety correlated with greater emphasis on alliance and less on techniques. Belief in alliance predicted lower encouragement of eating behavior change, while belief in techniques predicted more use of active CBT strategies. Education on actual therapy outcome factors and awareness of overvaluation is recommended. |
| Watt and Dickens | Community-based mealtime management for adolescents with anorexia nervosa: A qualitative study of clinicians' perspectives and experiences | 2018 | UK | CYP, community | AN | Qualitative interviews | MDT | Mealtime management involves technical, emotional, and uncertain aspects (e.g., supplement use). Clinicians found it valuable but emphasized the need for emotion regulation, formal training, and adherence to flexible protocols. |
| Webb and Schmidt | Facilitators and barriers to supporting young people with eating disorders during their transition to, and time at, university: An exploration of clinicians' perspectives | 2021 | UK | University transition |  | Qualitative interviews | MDT | Facilitators of ED treatment for university students included service flexibility, awareness, and carer support. Barriers included poor healthcare-university communication, limited GP involvement, inconsistent guidelines, and student ambivalence. Emphasized the value of carers and need for tailored, collaborative care between health and education sectors. |
| Webb, Dalton, Irish, Mercado, McCombie, Peachey, et al. | Clinicians' perspectives on supporting individuals with severe anorexia nervosa in specialist eating disorder intensive treatment settings during the COVID-19 pandemic | 2022 | UK | All | AN | Qualitative interviews | MDT | Key themes included intensive support, illness severity, hope, treatment matching, resource constraints, and carer burden. The importance of MDT collaboration and carer involvement was highlighted. Challenges were noted in managing complex needs and aligning treatment with patients' home contexts. More research is needed to compare IP to DP. |
| Welch | Recovered clinicians and the use of self-disclosure in the treatment of eating disorders | 2023 | USA |  |  | Qualitative interviews | LE | Clinicians with lived experience often disclosed their ED history to supervisors with little regret, though stigma deterred others. Disclosure was typically deliberate, weighing risks and benefits. Empathy was seen as a unique asset. Addressing workplace stigma and supporting safe, supervised self-disclosure may help reduce relapse risk. |
| Williams and Haverkamp | Eating Disorder Therapists' Personal Eating Disorder History and Professional Ethics: An Interpretive Description | 2015 | USA/Canada | all | All | Qualitative interviews | LE | LE therapists may face unique ethical vulnerabilities, especially early in their careers or recovery. Key issues include boundary management, therapist wellness, and challenges around disclosing personal history. Supportive supervision is essential, alongside clearer ethical guidance and decision-making models. |
| Wu and Chen | Nurses' perceptions on and experiences in conflict situations when caring for adolescents with anorexia nervosa: A qualitative study | 2021 | Taiwan | CYP | AN | Qualitative interviews | Nurses | Nurses struggled to form therapeutic relationships with adolescents with AN due to defensiveness, emotional triggers, and time constraints. They also faced difficulty addressing cognitive distortions and patient passivity. Ongoing support, in-service training, and trust-building are essential to improve care and therapeutic alliance. |
| Zaremba, Watson, Kan, Broadley, Partridge, Figuereido, et al. | Multidisciplinary healthcare teams' challenges and strategies in supporting people with type 1 diabetes to recover from disordered eating | 2019 | UK |  | T1DE | Qualitative interviews | MDT | Challenges included HCP feelings of incompetence, emotional burden, patient disengagement, and systemic issues like time pressure. Effective strategies included care plans, MDT clinics, and improving therapeutic alliance. Emphasized was the need for clear diagnostic criteria, training, and interdisciplinary resources for T1DE. |
| Zugai, Stein-Parbury and Roche | Therapeutic alliance, anorexia nervosa and the inpatient setting: A mixed methods study | 2018 | Australia | IP | AN | Mixed-methods | Nurses | Therapeutic alliance in AN inpatient care is challenged by the nature of the illness but can be strengthened by nurses’ close involvement and interpersonal skills. Supportive ward structures and adequate resources are essential for enabling meaningful nurse–patient interactions. |
| Zugai, Stein-Parbury and Roche | Dynamics of nurses' authority in the inpatient care of adolescent consumers with anorexia nervosa: A qualitative study of nursing perspectives | 2019 | Australia | CYP | AN | Qualitative interviews | Nurses | Young, inexperienced nurses were more vulnerable to challenges to their authority. Maintaining professional boundaries was key to preserving therapeutic use of power. Support and education are needed to help nurses navigate interactions that undermine authority, especially in settings requiring firm therapeutic leadership. |
