# Supplementary material for: A Scoping Review of Eating Disorder Clinicians' Experiences, Needs, Views and Wellbeing
Source: J Clin Psychol. 2025 Jun 16;81(10):903–17. doi: 10.1002/jclp.70005 (PMC12419239; doi:10.1002/jclp.70005)
Supplement: Supplementary file 2 — supp table 2 study risk of bias appraisals. [file JCLP-81-903-s002.docx]

Supplementary table 2. study risk of bias appraisal.

| Authors | Date | Study Design | Risk Appraisal | Justification |
| --- | --- | --- | --- | --- |
| Biang, A., Merlin‐Knoblich, C., and Lim, J. H. | 2024 | Qualitative Interview | Low | Appropriate design, description of data collection and analysis |
| Bommen, Nicholls and Billings | 2023 | Qualitative interview | Low | Appropriate design, description of data collection and analysis |
| Chang, Liao, Huang and S.Chen | 2023 | Qualitative interview | Low | Appropriate design, description of data collection and analysis |
| Coelho, J. S., Pardiwala, T., Marshall, S. K., Lam, P. Y., Grewal, S., Virani, A., ... and Geller, J. | 2024 | Mixed-methods survey | Low | Justified design, combination of qualitative and quantitative techniques, appropriate integration of data |
| Colleluori, Goria, Zillanti, Marucci and Dalla Ragione | 2021 | Mixed-methods survey | Low-moderate | Not enough description of qualitative analysis, quantitative measures not standardized |
| Corral-Liria, Alonso-Maza, Gonzalez-Luis, Fernandez-Pascual, Becerro-de-Bengoa-Vallejo and Losa-Iglesias | 2022 | Qualitative interview | Low | Appropriate design, description of data collection and analysis |
| Cowan | 2020 | Quantitative survey | Low | Appropriate description of data collection and analysis, justification of measures, no reporting bias |
| Crest, P., Vendlinski, S. S., Borges, R., Landsverk, J., & Accurso, E. C. | 2024 | Qualitative interview | Low | Appropriate design, description of data collection and analysis |
| Curry and Andriopoulou | 2023 | Qualitative interview | Low | Appropriate design, description of data collection and analysis |
| Daven, Hellzen and Haggstrom | 2022 | Qualitative interview | Low | Appropriate design, description of data collection and analysis |
| Davey, Arcelus and Munir | 2014 | Qualitative interview | Low-moderate | Little description of sampling and data collection, no reflexivity |
| de Vos, Netten and Noordenbos | 2016 | Qualitative questionnaire | Low | Appropriate design, description of data collection and analysis |
| Devery, Scanlan and Ross | 2018 | Mixed methods | Low | Justified design, combination of qualitative and quantitative techniques, appropriate integration of data |
| Downey, A. E., Odette, M., Sanders, A. E., Kuykendall, M., Saunders, E., Nagata, J. M., ... and Garber, A. K. | 2024 | Quantitative | Moderate | No procedure, sampling description, no standardized measures |
| Ferrucci, McPhillips, Lapane, Jesdale and Dube | 2023 | Qualitative interview | Low | Appropriate design, description of data collection and analysis |
| Ferrucci, Lapane, Jesdale, McPhillips and Dube | 2024 | Qualitative interview | Low | Appropriate design, description of data collection and analysis |
| Geller, Fernandes, Kelly, Samson and Srikameswaran | 2023 | Quantitative survey | Moderate | No procedure |
| Giannopoulos, E., and Hilsenroth, M. | 2024 | Quantitative survey | Low | Appropriate description of data collection and analysis, justification of measures, no reporting bias |
| Groth, Hilsenroth, Gold, Boccio and Tasca | 2020 | Quantitative survey | Low | Appropriate description of data collection and analysis, justification of measures, no reporting bias |
| Hage and Ro | 2020 | Quantitative survey | Low | Appropriate description of data collection and analysis, justification of measures, no reporting bias |
| Hage, Ro and Ro | 2021 | Quantitative survey | Low | Appropriate description of data collection and analysis, justification of measures, no reporting bias |
| Hage, Ro and Moen | 2017 | Qualitative interview | Low | Appropriate design, description of data collection and analysis |
| Hamama-Raz and Mazor | 2023 | Quantitative survey | Low | Appropriate description of data collection and analysis, justification of measures, no reporting bias |
| Harken, Maxwell, Hainline, Pollack and Roberts | 2017 | Qualitative interview | Low | Appropriate design, description of data collection and analysis |
| Honig | 2019 | Qualitative interview | Low | Appropriate design, description of data collection and analysis |
| Jing, E., Gregertsen, E., Chen, L., and Russell, J. | 2024 | Mixed methods | Low | Justified design, combination of qualitative and quantitative techniques, appropriate integration of data |
| King and Russon | 2023 | Qualitative interview | Low | Appropriate design, description of data collection and analysis |
| Kinnaird, Norton and Tchanturia | 2017 | Qualitative interview | Low | Appropriate design, description of data collection and analysis |
| Kinnaird, Norton and Tchanturia | 2018 | Qualitative interview | Low | Appropriate design, description of data collection and analysis |
| Kodua, Mackenzie and Smyth | 2020 | Qualitative interview | Low | Appropriate design, description of data collection and analysis |
| Kuehne, Hemmings, Phillips, Ince, Chounkaria, Ferraro, et al. | 2023 | Mixed-methods survey | Low | Justified design, combination of qualitative and quantitative techniques, appropriate integration of data |
| Lachal, Carretier, Prevost, Nadeau, Taddeo, Fortin, et al. | 2023 | Qualitative interview | Low | Appropriate design, description of data collection and analysis |
| Lennips, Peters, Meijboom, Nissen and Bunt | 2024 | Qualitative interview | Low | Appropriate design, description of data collection and analysis |
| Lev Ari, Safyon and Tuval-Mashiach | 2023 | Quantitative survey | Low | Appropriate description of data collection and analysis, justification of measures, no reporting bias |
| Levas-Luckman | 2016 | Qualitative interview | Low | Appropriate design, description of data collection and analysis |
| Levy | 2014 | Qualitative interview | Low | Appropriate design, description of data collection and analysis |
| Lloyd, Martin, Carney, Tattersall and Basu | 2022 | Mixed methods | N/A | Abstract only. |
| Lockertsen, Nilsen, Holm, Ro, Burger and Rossberg | 2020 | Qualitative interview | Low | Appropriate design, description of data collection and analysis |
| Love | 2018 | Qualitative interview | Low | Appropriate design, description of data collection and analysis |
| Macdonald, Kan, Stadler, De Bernier, Hadjimichalis, Le Coguic, et al. | 2018 | Qualitative interview | Low | Appropriate design, description of data collection and analysis |
| Matthews-Rensch, Young, Cutmore, Davis, Jeffrey and Patterson | 2023 | Qualitative interview | Low | Appropriate design, description of data collection and analysis |
| Mayer, Lemmer, Michelsen, Schrader, Friederich and Bauer | 2024 | Qualitative interview | Low | Appropriate design, description of data collection and analysis |
| McMaster, Wade, Franklin, Waller and Hart | 2022 | Quantitative survey | Low | Appropriate description of data collection and analysis, justification of measures, no reporting bias |
| McNicholas, OConnor, McNamara and O'Hara | 2018 | Mixed-methods | Low | Justified design, combination of qualitative and quantitative techniques, appropriate integration of data |
| Novack, Dufour, Picard, Taddeo, Nadeau, Katzman, et al. | 2023 | Mixed methods | Low | Justified design, combination of qualitative and quantitative techniques, appropriate integration of data |
| Oliverio, Steiger, St-Hilaire, Paquin-Hodge, Leloup, Israel, et al. | 2024 | Qualitative interviews | Low-moderate | No sampling description or reflexivity |
| Reas, Isomaa, Solhaug Gulliksen and Levallius | 2021 | Quantitative survey | Low | Appropriate description of data collection and analysis, justification of measures, no reporting bias |
| Retkiewicz | 2022 | Quantitative survey | Low | Appropriate description of data collection and analysis, justification of measures, no reporting bias |
| Ryu, Hamilton and Tarrant | 2022 | Qualitative interview | Low | Appropriate design, description of data collection and analysis |
| Shaw, Robertson and Ranceva | 2021 | Mixed methods | Moderate | No sampling description or procedure |
| Stocker, Rosenthal, Mesquida, Raynaud and Revet | 2022 | Qualitative interviews | Low | Appropriate design, description of data collection and analysis |
| Tragantzopoulou and Giannouli | 2023 | Qualitative interviews | Low | Appropriate design, description of data collection and analysis |
| Turner, Tatham, Lant, Mountford and Waller | 2014 | Quantitative survey | Low | Appropriate description of data collection and analysis, justification of measures, no reporting bias |
| Walsh, Davies, Pluckwell, Huffinley and Waller | 2019 | Quantitative survey | Low | Appropriate description of data collection and analysis, justification of measures, no reporting bias |
| Watt and Dickens | 2018 | Qualitative interviews | Low | Appropriate design, description of data collection and analysis |
| Webb and Schmidt | 2021 | Qualitative interviews | Low | Appropriate design, description of data collection and analysis |
| Webb, Dalton, Irish, Mercado, McCombie, Peachey, et al. | 2022 | Qualitative interviews | Low | Appropriate design, description of data collection and analysis |
| Welch | 2023 | Qualitative interviews | Low | Appropriate design, description of data collection and analysis |
| Williams and Haverkamp | 2015 | Qualitative interviews | Low | Appropriate design, description of data collection and analysis |
| Wu and Chen | 2021 | Qualitative interviews | Low | Appropriate design, description of data collection and analysis |
| Zaremba, Watson, Kan, Broadley, Partridge, Figuereido, et al. | 2019 | Qualitative interviews | Low | Appropriate design, description of data collection and analysis |
| Zugai, Stein-Parbury and Roche | 2018 | Mixed-methods | Low | Justified design, combination of qualitative and quantitative techniques, appropriate integration of data |
| Zugai, Stein-Parbury and Roche | 2019 | Qualitative interviews | Low | Appropriate design, description of data collection and analysis |
